# Nanomolar Affinity Host‐Dye Reporter Pairs from Fluorescently Labeled Oligoarginine Peptides and *p*‐Sulfonatocalix[4]arene

**DOI:** 10.1002/cbic.202500782

**Published:** 2025-11-26

**Authors:** Aparna Pramanik, Mohammad A. Alnajjar, Tristan Wegner, Andreas Hennig

**Affiliations:** ^1^ Center for Cellular Nanoanalytics (CellNanOs) and School of Biology/ Chemistry Universität Osnabrück 49069 Osnabrück Germany

**Keywords:** assays, chemosensors, fluorescent dyes, host–guest chemistry, oligoarginines

## Abstract

Oligoarginine peptides are of interest as cell‐penetrating peptides (CPPs) and play important roles in gene regulation and immune response. Complexation of oligoarginine peptides by the supramolecular host *p‐*sulfonatocalix[4]arene (CX4) is well‐established and has led to the use of CX4 derivatives as effective counterion activators for arginine‐rich CPPs and to the application of CX4 in peptide sensing. Herein, a systematic binding study between oligoarginine peptides with CX4 is reported. The results show that the binding affinity increases with increasing peptide length from ≈10^4 ^M^–1^ for the peptide H‐Arg‐Arg‐OH to nanomolar affinities for peptides with more than four arginine residues. Within the series, C‐terminal carboxamides show higher affinities than the respective carboxylates. In addition, the influence of fluorescent dye labeling is investigated with sulforhodamine B (SRB) and fluorescein (FL) dye labels. Efficient fluorescent quenching is observed after complexation of the labeled peptides by CX4, which prompted the exploration of the complexes as reporter pairs for chemosensing applications. The results suggest that the combination of fluorescently labeled oligoarginine peptides with CX4 affords a modular platform of host‐dye reporter pairs for the detection of polycationic peptides with tailorable excitation and emission wavelengths and tailorable binding affinity down to the nanomolar affinity range.

## Graphical abstract

The dependence of the binding affinity of oligoarginine peptides to *p*‐sulfonatocalix[4]arene (CX4) on peptide length, C‐terminal functionality, and fluorescent labeling is reported. The fluorescent peptides can be excited in the visible range with common lasers and show nanomolar affinity and efficient quenching upon CX4 binding, which enables their use as modular host‐dye reporter pairs for peptide sensing.

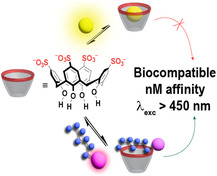

## 1 Introduction

The binding of biomolecules by synthetic supramolecular receptors in water paves the way for numerous applications, for example in medicine, bioanalysis, nanobiotechnology, molecular biology, or drug delivery.^[^ ^1^, ^2^, ^3^, ^4^, ^5^, ^6^, ^7^, ^8^, ^9^, ^10^, ^11^, ^12^, ^13^, ^14^, ^15^, ^16^, ^17^, ^18^, ^19^ ^]^ Among the different biomolecules, peptides are particularly intriguing since they play numerous roles in living organisms. Binding of peptides by supramolecular receptors has been shown to induce peptide folding,^[^ ^20^, ^21^, ^22^, ^23^, ^24^ ^]^ influence peptide aggregation and protein binding,^[^ ^25^, ^26^, ^27^, ^28^, ^29^, ^30^, ^31^ ^]^ induce peptide and protein dimerization,^[^ ^32^, ^33^, ^34^ ^]^ affect membrane partitioning and transport,^[^ ^35^, ^36^, ^37^, ^38^, ^39^, ^40^, ^41^, ^42^, ^43^ ^]^ inhibit enzymatic reactions,^[^ ^44^ ^]^ and enable pull‐down assays,^[^ ^45^ ^]^ affinity labeling,^[^ ^46^ ^]^ and peptide beacons.^[^ ^47^ ^,^ ^48^ ^]^ Established supramolecular receptors for peptides include, for example, cucurbiturils, which are prone to bind hydrophobic amino acids or amino acid sequences at the *N*‐terminus with astoundingly high micromolar to picomolar binding affinities,^[^ ^20^ ^,^ ^21^ ^,^ ^32^, ^33^, ^34^ ^,^ ^43^, ^44^, ^45^ ^,^ ^49^, ^50^, ^51^, ^52^, ^53^ ^]^ while deep cavitands,^[^ ^17^, ^18^, ^19^ ^]^ molecular tweezers and clips,^[^ ^29^, ^30^, ^31^ ^,^ ^54^ ^]^ and calixarenes,^[^ ^12^, ^13^, ^14^, ^15^, ^16^ ^,^ ^35^, ^36^, ^37^, ^38^, ^39^, ^40^, ^41^, ^42^ ^,^ ^46^ ^]^ have been used for the complexation of cationic amino acid residues, such as lysine and arginine residues and their methylated derivatives.

Polycationic peptides play vital roles in the body. They are involved in transporting molecules across membranes, regulate gene expression by binding nucleic acids, and contribute to immune responses. One particular class of polycationic peptides are cell‐penetrating peptides (CPPs), which are of interest because they promote the cellular uptake of a conjugated cargo and can thus serve as delivery vectors for therapeutic drugs or diagnostic imaging agents.^[^ ^55^, ^56^, ^57^, ^58^, ^59^, ^60^, ^61^, ^62^, ^63^, ^64^, ^65^ ^]^ Given their biological significance and clinical relevance, it is important to develop efficient methods for their analysis and detection.^[^ ^66^, ^67^, ^68^ ^]^

The supramolecular host *p*‐sulfonatocalix[4]arene (CX4) is well‐known to bind to the cationic amino acids lysine and arginine.^[^ ^69^, ^70^, ^71^, ^72^, ^73^, ^74^, ^75^ ^]^ CX4 has been used in fluorescent host‐dye reporter pairs as a molecular recognition element for the detection of polycationic peptides,^[^ ^15^ ^,^ ^16^ ^,^ ^76^ ^]^ and amphiphilic *p*‐sulfonatocalix[n]arene derivatives have been recently established as highly efficient counterion activators for oligoarginine CPPs.^[^ ^35^, ^36^, ^37^, ^38^, ^39^, ^40^ ^]^ The binding of arginine‐containing peptides is of fundamental importance for these applications and the reported binding affinities reach from the millimolar range for amino acids and short peptides to subnanomolar affinities for longer oligoarginines.^[^ ^40^ ^,^ ^74^ ^,^ ^75^ ^]^ Despite the significant interest, a systematic binding study of CX4 with short oligoarginines is so far lacking.

**Figure 1:**
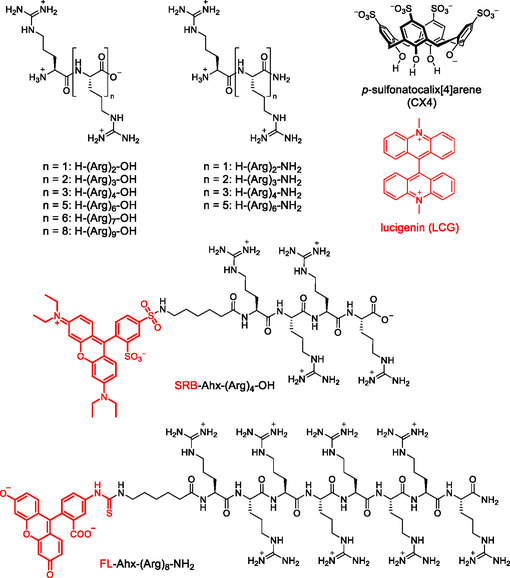
Chemical structures of the supramolecular host CX4, the fluorescent dye LCG, and the unlabeled and fluorescently labeled peptides investigated in this study. Shown is the predominant protonation state of the compounds at neutral pH.

Herein, we provide a systematic investigation of the binding affinities of oligoarginine peptides with two to nine arginine residues to CX4 (**Figure** **1**). We use fluorescence displacement titrations and isothermal titration calorimetry (ITC) to investigate the influence of peptide length and of C‐terminal amidation. We also include two fluorescently labeled peptides to test for the influence of dye labels on the binding affinity and report quenching of the dyes by binding of the peptides to the CX4 host. We further demonstrate that the combination of the CX4 host with the fluorescently labeled peptides affords a modular chemosensor system with tunable binding affinity and tunable excitation and emission wavelengths for the fluorescence‐based detection of CPPs and other polycationic peptides.

## 2 Results and Discussion

### 2.1 Binding Affinity of Oligoarginine Peptides to CX4

To investigate the influence of oligomer length of the arginine peptides on the binding affinity with CX4, a series of oligoarginine peptides with two to nine arginine residues with C‐terminal carboxylic acid and carboxamide groups was synthesized by solid‐phase peptide synthesis (see Supporting Information). Specifically, we included peptides of the following sequences: H‐(Arg)_*n*_‐OH with *n* = 2, 3, 4, 6, 7, and 9 as well as H‐(Arg)_*n*_‐NH_2_ with *n* = 2, 3, 4, and 6 (see Figure 1).

**Figure 2:**
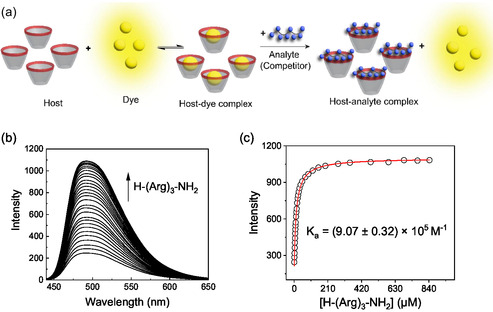
a) Schematic representation of the indicator displacement principle. The supramolecular host (here: CX4) complexes a fluorescent dye leading to a non‐fluorescent host–guest complex. Addition of another molecule (i.e., an analyte or a competitor) that can also bind to the host leads to dye displacement and to an increased fluorescence. The principle can be used in competitive binding titrations to determine the binding affinity of the competitor or it can be applied as a sensor to detect and quantify an analyte in an indicator displacement assay (IDA). b) Competitive fluorescence titration (*λ*_ex_ = 369 nm) with 0.5 µM LCG, 1 µM CX4, and varying concentrations of H‐(Arg)_3_‐NH_2_ in 10 mM NaH_2_PO_4_, pH 7.2, 25 °C. c) Respective titration curve (*λ*_em_ = 492 nm) with fitted line.

The apparent binding affinities (*vide infra*) of the selected peptides to CX4 were then quantitatively analyzed using fluorescence competitive binding titrations (**Figure** **2**) with the fluorescent dye lucigenin (LCG). LCG binds to CX4 with binding constants on the order of 10^7 ^M^−1^, and LCG is efficiently quenched upon complexation (Figure S23, Supporting Information).^[^ ^77^ ^]^ Addition of the oligoarginine peptides to the CX4/LCG host–guest complex displaces LCG from CX4, which leads to a fluorescence increase and enables the determination of the oligoarginine peptide binding constants (Figure S24–S32, Supporting Information). The results (**Table** **1**) show that the binding affinity of the oligoarginine peptides to CX4 increases with increasing length of the peptides. This is attributed to the increased number of arginine residues, providing more contact points for electrostatic interactions between the negatively charged sulfonate groups of the calixarene and the positively charged guanidinium groups of arginine. The length‐dependent trend in binding affinities was consistent for both types of carboxy termini, whereas the peptides with C‐terminal amide groups exhibited a higher binding affinity than their carboxylic acid‐terminated counterpart. An additional notable observation was that the binding constants of the H‐(Arg)_(*n*−1)_‐NH_2_ peptides were closely comparable to those of their corresponding H‐(Arg)_*n*_‐OH analogs, that is, the binding affinity of H‐(Arg)_2_‐NH_2_ was comparable to the binding affinity of H‐(Arg)_3_‐OH, the binding affinity of H‐(Arg)_3_‐NH_2_ was comparable to the binding affinity of H‐(Arg)_4_‐OH, and so on. This suggests that the number of net positive charges on the oligoarginine peptides has a significant influence on their binding affinity to CX4, although other factors such as the peptide length and hydrophobicity may contribute as well.^[^ ^43^ ^]^ Collectively, the binding affinities followed the order: H‐(Arg)_2_‐OH < H‐(Arg)_2_‐NH_2_ ≈ H‐(Arg)_3_‐OH < H‐(Arg)_3_‐NH_2_ ≈ H‐(Arg)_4_‐OH < H‐(Arg)_4_‐NH_2_ ≈ H‐(Arg)_6_‐OH < H‐(Arg)_6_‐NH_2_. As a rule of thumb, the binding affinity increases for shorter peptides by almost a factor of 10 for each additional positive charge, whereas the effect levels off at longer oligoarginine peptide lengths.

**Table 1:** Binding affinity of oligoarginine peptides to CX4.

Peptidea)	*K*_a_ [M^–1^]	
	FLb)	ITC
H‐(Arg)_2_‐OH	(1.54 ± 0.28) × 10^4^	(3.96 ± 0.52) × 10^4^
H‐(Arg)_2_‐NH_2_	(8.60 ± 0.40) × 10^5^	(5.36 ± 0.70) × 10^5^
H‐(Arg)_3_‐OH	(4.43 ± 0.18) × 10^5^	(4.16 ± 0.26) × 10^5^
H‐(Arg)_3_‐NH_2_	(9.07 ± 0.32) × 10^5^	(2.18 ± 0.12) × 10^6^
H‐(Arg)_4_‐OH	(2.40 ± 0.04) × 10^6^	(1.43 ± 0.27) × 10^6^
H‐(Arg)_4_‐NH_2_	(1.05 ± 0.08) × 10^7^	(1.92 ± 0.75) × 10^7^
H‐(Arg)_6_‐OH	(2.43 ± 0.10) × 10^7^	(1.39 ± 0.49) × 10^7^
H‐(Arg)_6_‐NH_2_	(1.44 ± 0.55) × 10^8^	(0.62 ± 0.05) × 10^8^
H‐(Arg)_7_‐OH	(7.18 ± 0.48) × 10^7^ c)	n.d.d)
H‐(Arg)_9_‐OH	(1.82 ± 0.42) × 10^8^	n.d.d)

a) Apparent binding affinities, *K*_a_, in 10 mM NaH_2_PO_4_, pH 7.2 at 25 °C;

b) Determined by competitive titration with CX4/LCG;

c) In 10 mM NaH_2_PO_4_, pH 7.2; taken from ref. [43];

d) n.d. = not determined.

The binding constants were further confirmed by ITC measurements (Figure S33–S36, Supporting Information) and the results were in good agreement with the values obtained from the fluorescence displacement titrations (Table 1). During the analysis of the ITC binding titration curves, we noted that the data obtained with the shorter peptides could be fitted well using a one‐site binding model, whereas the ITC titration data of the longer peptides was better described by a two‐site binding model indicating the presence of multiple interaction sites or modes of binding. This observation suggests that while shorter peptides likely interact with calixarenes in a 1:1 host‐to‐guest stoichiometry, the longer peptides may engage in complexes with higher host‐to‐guest ratios. The increased binding stoichiometry is attributed to the increased length and flexibility of the peptide chains, allowing for additional interaction points with the calixarene host. The second binding site of the longer peptides was, however, three orders of magnitude weaker (≈10^4 ^M^–1^) than the first binding site (>10^7 ^M^–1^). Binding of a second CX4 host to the peptides was thus negligible in the fluorescence displacement titrations, which were conducted at much lower concentrations (≤1 µM) than the ITC titrations (20 to 100 µM), and which could be all fitted well with a 1:1 binding model.

### 2.2 Fluorescently Labeled Peptides

In addition to the series of unlabeled oligoarginine peptides, two different fluorescent peptides were synthesized and studied (Figure 1). We have selected fluorescein‐5‐isothiocyanate (FITC) and sulforhodamine B (SRB) chloride as fluorescent labels, because they are commercially available labeling reagents with absorption and emission wavelengths that are compatible with common laser excitation sources. The dyes were N‐terminally introduced via a 6‐aminohexanoic acid (Ahx) linker affording a FITC‐labeled octaarginine peptide and a SRB‐labeled tetraarginine peptide (see Supporting Information for details).

The FITC‐labeled peptide was synthesized by N‐terminal extension of the peptide chain during solid phase peptide synthesis with Fmoc‐protected 6‐aminohexanoic acid (Fmoc‐Ahx‐OH) and subsequent reaction with two equivalents of the 5‐isomer of FITC. With SRB, an alternative reaction route was followed, namely SRB sulfonyl chloride was first reacted with 6‐aminohexanoic acid methyl ester (H‐Ahx‐OMe) in solution and the ester was hydrolyzed to afford SRB‐Ahx‐OH, which was then N‐terminally coupled to the peptide chain during solid phase peptide synthesis. This alternative procedure was pursued for two reasons: First, direct coupling of SRB sulfonyl chloride to the Ahx linker on solid phase gave only very low yields, and second, commercially available SRB sulfonyl chloride is a mixture of two isomers with the sulfonyl chloride functional group in the 2‐ and 5‐position. Purification and identification of the two isomers according to literature methods^[^ ^78^ ^]^ was best achieved with H‐Ahx‐OMe and all subsequent reactions were then performed with the purified 5‐isomer.

**Figure 3:**
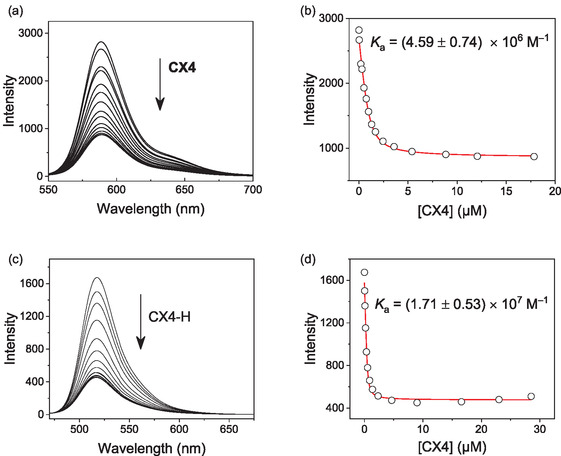
a) Fluorescence titration of 1.0 µM SRB‐Ahx‐(Arg)_4_‐OH (*λ*_ex_ = 525 nm) with varying concentrations of CX4 in 10 mM NaH_2_PO_4_, pH 7.2, 25 °C. b) Respective titration curve (*λ*_em _= 588 nm) with fitted line. c) Fluorescence titration of 0.5 µM FL‐Ahx‐(Arg)_8_‐NH_2_ (*λ*_ex_ = 420 nm) with varying concentrations of CX4 in 10 mM NaH_2_PO_4_, pH 7.2, 25 °C. d) Respective titration curve (*λ*_em _= 518 nm) with fitted line.

Binding of the fluorescent peptides to CX4 was investigated by direct fluorescence titrations, which revealed fluorescence quenching upon host–guest complex formation (**Figure** **3**). The fluorescence of the SRB‐labeled peptide at 588 nm decreased by a factor of 3.2 and the fluorescence of the FITC‐labeled peptide at 518 nm decreased by a factor of 3.7. The respective titration plots showed nanomolar affinity with binding constants of 4.6 × 10^6^ and 1.7 × 10^7 ^M^−1^, respectively (**Table** **2**). Compared to the unlabeled peptides, the binding affinity of the SRB‐labeled peptide was slightly increased, while the affinity of the FITC‐labeled peptide was decreased (cf. Table 1 and 2). This trend is in agreement with the net charge of the fluorescent dyes. The two negative charges on the FITC label lead to an electrostatic repulsion between the peptide and the negatively charged CX4 host, which reduces the binding affinity. The zwitterionic SRB label has consequently a much lower influence and the slightly increased affinity may originate from a more specific interaction between the positively charged nitrogen atoms on the xanthene group of SRB and the negatively charged CX4 or from a hydrophobic contribution of the aromatic SRB dye with the CX4 host cavity.

**Table 2:** Binding affinities and fluorescence changes of fluorescently labeled peptides upon binding to CX4.

Peptidea)	*K*_a_ [M^–1^]	Quenchingfactorb)
SRB‐Ahx‐(Arg)_4_‐OH	(4.59 ± 0.74) × 10^6^	3.2
FL‐Ahx‐(Arg)_8_‐NH_2_	(1.71 ± 0.53) × 10^7^	3.7

a) In 10 mM NaH_2_PO_4_, pH 7.2 at 25 °C

b) Ratio of fluorescence intensities in absence of CX4 and after full complexation.

A potential reason for the observed fluorescence quenching is photoinduced electron transfer (PET) from the electron‐rich CX4 to the excited state of the fluorescent dye. Calculation of the Gibb's free energy of PET (*ΔG*_PET _= *E*_ox_ *− E*_red_ *− E*_0,0_)^[^ ^79^ ^]^ with the reported oxidation potential (*E*_ox_) of CX4 (+0.95 V versus Ag/AgCl),^[^ ^77^ ^]^ the reduction potential (*E*_red_) of fluorescein (–0.88 V versus Ag/AgCl),^[^ ^80^ ^]^ and the singlet excitation energy (*E*_0,0_) of FITC (≈506 nm, i.e., 2.45 eV) indicates that acceptor‐excited PET is indeed thermodynamically favorable (*ΔG*_PET _= −0.60 eV). The same applies to quenching of SRB by CX4, which gave *ΔG*_PET _= −0.25 eV.^[^ ^81^ ^]^ Furthermore, the fact that the shape and position of the fluorescence bands of both dyes do not change is in accordance with a PET quenching mechanism (Figure 3). Another notable observation is that the experimentally determined quenching factors are small compared to other host‐dye reporter pairs based on PET.^[^ ^77^ ^]^ This points toward different conformations of the peptide during the excited‐state lifetime of the fluorescent dye, in which only a fraction of the dye population is sufficiently close to CX4 to afford electron transfer rates that can outcompete the radiative decay rate of the dye.^[^ ^82^ ^]^

### 2.3 Fluorescence‐Based Sensing

**Figure 4:**
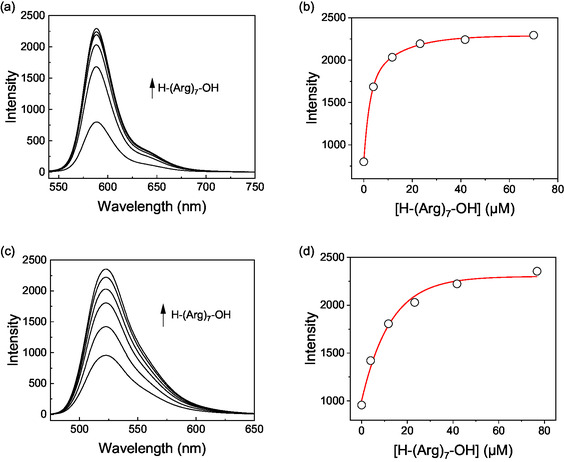
Detection of the peptide H‐(Arg)_7_‐OH in an indicator displacement assay using the host CX4 in combination with a,b) the rhodamine‐based peptide SRB‐(Arg)_4_‐OH (*λ*_ex_ = 525 nm; *λ*_em_ = 588 nm) or c,d) the fluorescein‐based peptide FL‐(Arg)_8_‐NH_2_ (*λ*_ex_ = 460 nm; *λ*_em_ = 523 nm) as a host‐dye reporter pair. Experiments were conducted in 10 mM NaH_2_PO_4_, pH 7.2 at 25 °C with 1 µM and 1 µM CX4.

The fluorescence quenching upon binding of the fluorescently labeled peptides to CX4 enables their application in CX4‐based chemosensors according to the indicator displacement assay (IDA) principle.^[^ ^83^ ^]^ IDA involves a fluorescent guest molecule, also termed an indicator, which is reversibly bound to a synthetic receptor host molecule leading to fluorescence quenching or enhancement. In the presence of a competitively binding molecule, the fluorescent guest is displaced from the host resulting in a change in its fluorescence. The principle is reminiscent of a competitive binding titration (Figure 2), but focuses on the analytical detection of the competitor (i.e., the analyte) rather than on the determination of its binding constant. In IDA, the signal output of the fluorescent dye is directly correlated with the molecular recognition event by the host, which enables analyte detection and quantification. The combination of a fluorescent dye and a host molecule that is suitable to be employed in an IDA is also termed a “host‐dye reporter pair”.^[^ ^76^ ^,^ ^84^ ^]^ To illustrate the utility of the host–guest complexes of SRB‐Ahx‐(Arg)_4_‐OH and FL‐Ahx‐(Arg)_8_‐NH_2_ with CX4 as host‐dye reporter pairs, we have selected the heptaarginine peptide H‐(Arg)_7_‐OH as a model analyte. Heptaarginine is of interest, because it is a CPP, it is a prototypical substrate for the enzyme trypsin, and it has been used to probe translocation of peptides through membrane protein pores.^[^ ^16^ ^,^ ^40^ ^,^ ^76^ ^]^ The addition of the heptaarginine analyte resulted in an immediate fluorescence increase, which demonstrates that the analyte can be reliably detected at low micromolar concentrations with both host‐dye reporter pairs (**Figure** **4**).

Established fluorescent dyes for the CX4 host include, for example, LCG,^[^ ^77^ ^]^ diazabicyclo[2.2.2]oct‐2‐ene (DBO), 4‐hydroxy‐10‐methyl pyranoflavylium, 1‐methyl‐6‐alkoxy‐quinolinium, or 1‐alkyl‐6‐methoxy‐quinolinium.^[^ ^85^, ^86^, ^87^ ^]^ Although the respective reporter pairs have proven useful,^[^ ^76^ ^]^ they also share some typical shortcomings. For example, many established dyes are excited in the UV wavelength region, which causes a high background fluorescence in cellular and medical imaging. Moreover, many fluorescent dyes are not sufficiently stable under prolonged exposure times in laser‐based applications such as confocal fluorescence microscopy imaging and the dyes consequently suffer from photobleaching.^[^ ^15^ ^]^ Lastly, the low binding affinity of many fluorescent dyes to the CX4 host limits their utility. In this context, it is noteworthy that the binding affinities reported herein represent only apparent affinities, because the influence of the Na^+^ cations in the employed phosphate buffer has been neglected. Monovalent and divalent alkali and earth alkali cations are known as competitive binders to CX4,^[^ ^88^ ^,^ ^89^ ^]^ which means at the one hand that the true binding affinities of the peptides are probably larger than the values reported herein, but also that typical low‐affinity host‐dye reporter pairs will dissociate in competitive environments such as body fluids, cells, and tissues due to high concentrations of salts.

In comparison with established host‐dye reporter pairs, the combination of the fluorescently labeled peptides with the CX4 host presented herein offers a unique perspective to rectify the mentioned shortcomings. First, the binding affinity of the peptide to the CX4 host can be tailored through the peptide length and amino acid sequence such that the host‐dye reporter pair is stable in physiological conditions, but remains sufficiently responsive toward an analyte.^[^ ^90^ ^]^ Second, the use of peptide‐dye conjugates presents a modular design that includes the possibility to select a dye label to meet specific requirements for excitation and emission wavelengths and stability, in which binding to the CX4 host is expected to lead to a significant change in the fluorescent signal. Lastly, a high biocompatibility is expected due to the combination of a peptide with fluorescent dyes established in bioimaging.

Overall, host–guest complexes of fluorescently labeled peptides with CX4 combine several attractive features of an ideal reporter pair for sensing applications and offer the ability to detect the presence of analytes through a clear change in fluorescence at modularly tailorable wavelengths. The customizable use of fluorescent dyes could be extended to the far‐red or NIR region for low background and deep tissue imaging and the use of dyes with ratiometric output signals could confer robustness to the read‐out. In practical terms, we propose that host‐dye reporter pairs with CX4 and fluorescent peptides will become an efficient tool for biosensing that could be used to monitor peptide transport, assess peptide stability, study peptide‐ligand interactions, or evaluate real‐time cellular uptake and track molecular transport in cells in applications such as bioassays, diagnostics, or environmental monitoring where detecting low concentrations of cationic peptides is desirable.^[^ ^37^ ^,^ ^38^ ^]^

## 3 Conclusion

In conclusion, we have systematically investigated the dependence of the binding affinity of oligoarginine peptides to the supramolecular host *p*‐sulfonatocalix[4]arene (CX4) on peptide length and C‐terminal modifications. The study was extended to include peptide modifications with fluorescent dyes, which were N‐terminally attached via a 6‐aminohexanoic acid linker. The latter revealed that sulforhodamine B and fluorescein dyes are efficiently quenched by binding of the labeled peptides to the calixarene host. This serendipitous finding prompted us to demonstrate the utility of the supramolecular host–guest complexes of CX4 with fluorescent peptides as host‐dye reporter pairs in sensing applications. The novel peptide‐based host‐dye reporter pairs are compatible with common laser excitation sources such as Ar lasers with 488 nm emission for excitation of fluorescein dyes and He‐Ne lasers with 543 nm emission for excitation of rhodamine dyes. We propose that the strategy provides a modular platform of host‐dye reporter pairs for the detection of polycationic peptides with tailorable binding affinity and excitation and emission wavelengths.

## Supporting Information

The authors have cited additional references within the Supporting Information.^[^ ^91^, ^92^, ^93^, ^94^, ^95^, ^96^, ^97^, ^98^, ^99^, ^100^ ^]^

## Conflict of Interest

The authors declare no conflict of interest.

## Supporting information

Supplementary Material

## Data Availability

The data that support the findings of this study are available from the corresponding author upon reasonable request.
