# Supplementary material for: Nanomolar Affinity Host‐Dye Reporter Pairs from Fluorescently Labeled Oligoarginine Peptides and p‐Sulfonatocalix[4]arene
Source: Chembiochem. 2025 Nov 26;27(1):e202500782. doi: 10.1002/cbic.202500782 (PMC12781158; doi:10.1002/cbic.202500782)
Supplement: Supplementary file 1 — Supplementary Material [file CBIC-27-e202500782-s001.pdf]

Supporting Information

for

Nanomolar Affinity Host-Dye Reporter Pairs from Fluorescently Labelled Oligoarginine Peptides and *p*-Sulfonatocalix[4]arene

Aparna Pramanik, Mohammad A. Alnajjar, Tristan Wegner, Andreas Hennig*

Center for Cellular Nanoanalytics (CellNanOs) and Department of Biology and Chemistry
Universität Osnabrück, 49069 Osnabrück (Germany); E-mail: andreas.hennig@uni-osnabrueck.de

Table of Contents

Abbreviations 2
Materials and Methods 2
Materials 2
Instrumentation 2
Preparation of MS Samples for MALDI 2
Synthesis 3
Synthesis of SRB-Ahx-OH 3
Oligoarginine Peptide Synthesis 11
Synthesis of Fluorescently Labelled Peptides 14
Determination of Binding Constants 18
Determination of Peptide Concentrations 18
Competitive Titrations with Oligoarginine Peptides 18
Isothermal Titration Calorimetry (ITC) 22
References 25

Abbreviations

$CDCl_3$: chloroform-d; CD_3OD: methanol-d4; CX4: *p*-sulfonatocalix[4]arene; DCM: dichloromethane; DHB: 2,5-dihydroxy benzoic acid; DIPEA: diisopropylethylamine; DMF: *N,N*-dimethylformamide; FITC: fluorescein-5-isothiocyanate; Fmoc: 9-fluorenyl-methyloxycarbonyl; HCCA: α-cyano-4-hydroxycinnamic acid; HCTU: 2-(6-chloro-1-H-benzotriazole-1-yl)-1,1,3,3-tetramethylaminium hexafluorophosphate; LCG: lucigenin; RP-HPLC: reversed-phase high-performance liquid chromatography; SPPS: solid-phase peptide synthesis; SRB: sulforhodamine-B; TFA: trifluoroacetic acid.

Materials and Methods

Materials

Reagents for buffer preparation and analytical measurements were from Fluka or Fisher scientific and of highest purity available. The two peptides H-$(Arg)_2$-OH and H-$(Arg)_3$-OH were purchased from Bachem (Bubendorf, Switzerland) and had a purity >95%. H-$(Arg)_7$-OH and H-$(Arg)_9$-OH were custom-synthesized by NovoPro Bioscience Inc. (Shanghai, China). Reagents for peptide synthesis, Fmoc-Arg(Pbf)-OH, FITC, sulforhodamine-B-sulfonyl chloride, HCTU, Wang Resin, and Rink amide resin were from Novabiochem, and Fmoc-Ahx-OH was from abcr GmbH (Germany). DIPEA, piperidine, and TFA were from Carl ROTH (Germany). Anisole, thioanisole, 1,2-ethanedithiol, LCG, and *p*-sulfonatocalix[4]arene were purchased from Sigma-Aldrich. Millipore water was prepared by a Merck Millipore simplicity UV water Purification system providing ultrapure water type 1 with ultralow TOC levels (≥18.2 MΩ cm, ≤5 ppb TOC). Buffers were prepared from the acid form by addition of NaOH to adjust the pH at 25 °C, such that the 10 mM NaH_2PO_4, pH 7.2 buffer contained ca. 16.5 mM Na^+.

Instrumentation

^{1}H and ^{13}C NMR spectra were measured on a Bruker AVIII 500 MHz NMR. ^{1}H NMR in CD_3OD:$CDCl_3$ (1:1) were referenced to the signal of $CHCl_3$ at 7.90 ppm and ^{13}C NMR were referenced to $CDCl_3$ at 79.44 ppm. Fluorescence measurements were performed on a Jasco FP-8300 spectrofluorimeter and UV/Vis absorption measurements were performed on a Jasco V-750 spectrophotometer (Pfungstadt, Germany). pH measurements were performed with a WTW pH 526 pH meter with a WTW SenTix Mic electrode. All the mass data were measured by MALDI Ultraflextreme II (Bruker Daltonic). For HPLC, an Agilent Technologies 1100 model was used with a Büchi PrepPure C18 column (100 Å, 5 μm, 250 × 4.6 mm) using a gradient of water and acetonitrile (both with 0.1% TFA). For lyophilization, an Alpha 1-2 LSCbasic (Martin Christ Gefriertrocknungsanlagen GmbH, Germany) was used.

Preparation of MS Samples for MALDI

All unlabelled peptides and SRB-Ahx-$(Arg)_4$-OH were dissolved in Millipore water and measured with HCCA matrix. FL-Ahx-$(Arg)_8$-NH_2 dissolved in Millipore water was measured with DHB matrix.

Synthesis

Synthesis of SRB-Ahx-OH

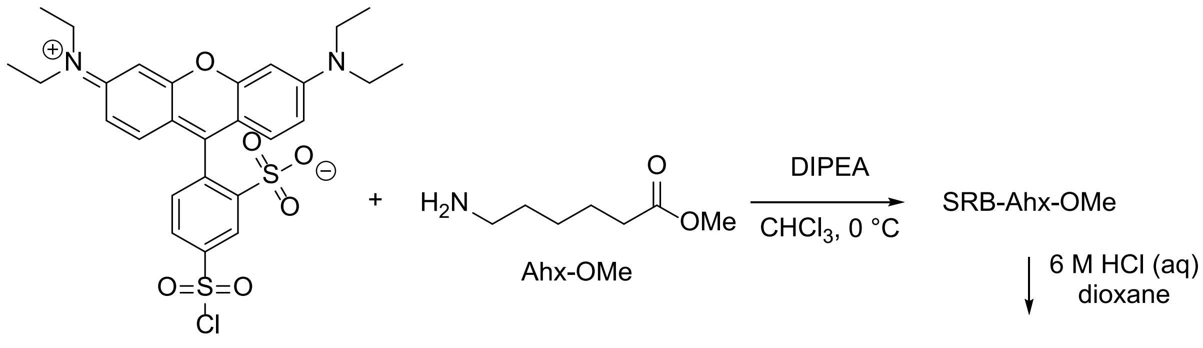

sulforhodamine B sulfonyl chloride
(aka lissamine rhodamine B sulfonyl chloride)
SRB chloride

SRB-Ahx-OMe. To a solution of methyl 6-aminohexanoate hydrochloride (Ahx) (0.188 g, 1.04 mmol) and 0.722 ml of diisopropylethylamine in 25 ml of chloroform was added sulforhodamine-B (SRB) sulfonyl chloride (0.300 g, 0.519 mmol) in small portions at 0 °C over a period of 15 minutes. Then, the reaction mixture was stirred at room temperature for overnight. The reaction was monitored by reverse phase thin layer chromatography. After that, the reaction mixture was washed three times with 50 ml of water. The organic layer was separated, dried over anhydrous sodium sulfate and concentrated under reduced pressure. A dark, purple solid was obtained. The solid was dissolved in methanol and any insoluble material was separated by centrifugation. The supernatant was concentrated and the solution was purified by flash column chromatography using Pure C-850 Flash (Büchi) with the silica 60 A (particle size 60-200 micron) and DCM/methanol (90:10). The purification gave two compounds with different R_f values (R_f = 0.38 and 0.30 in 10% MeOH/DCM) yet identical mass with a total yield of 73%. As commercial SRB sulfonyl chloride is a mixture of two sulfonyl chloride isomers, the presumed products are the “para”- and “ortho-isomers” shown below in **Figure S1**.[1]

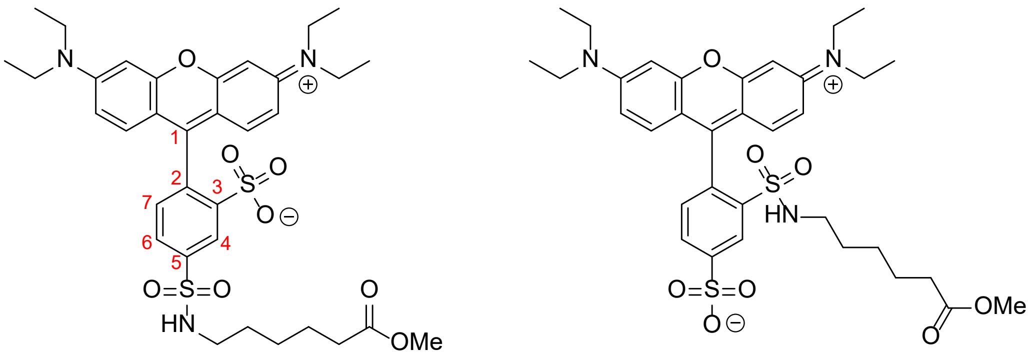

5-sulfonamide "para-isomer"

3-sulfonamide "ortho-isomer"

Figure S1. Presumed isomers of SRB-Ahx-OMe obtained during the synthesis.

Assignment of the two isomers was performed according to a literature method, which was established for SRB sulfonamides.[2] The method is based on the pH-dependent cyclization of

SRB sulfonamides, which converts the 3-sulfonamide (ortho-isomer) from a colored, open form into a colorless spirosultam form under basic conditions (**Figure S2**), whereas the 5-sulfonamide (para-isomer) cannot undergo such a cyclization reaction and remains therefore colored.

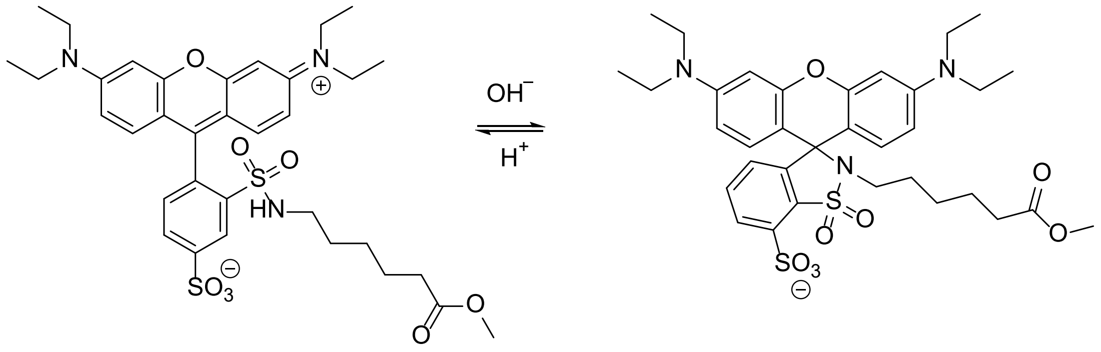

Figure S2. pH-dependent cyclization of the 3-sulfonamide isomer.

Measurement of the absorption spectra of both compounds in 25% EtOH aqueous solution at acidic and alkaline pH unambiguously identified the compound with an R_f value of 0.30 as the 3-sulfonamide isomer through a disappearance of the absorption band at 569 nm due to spirosultam formation (**Figure S3**).

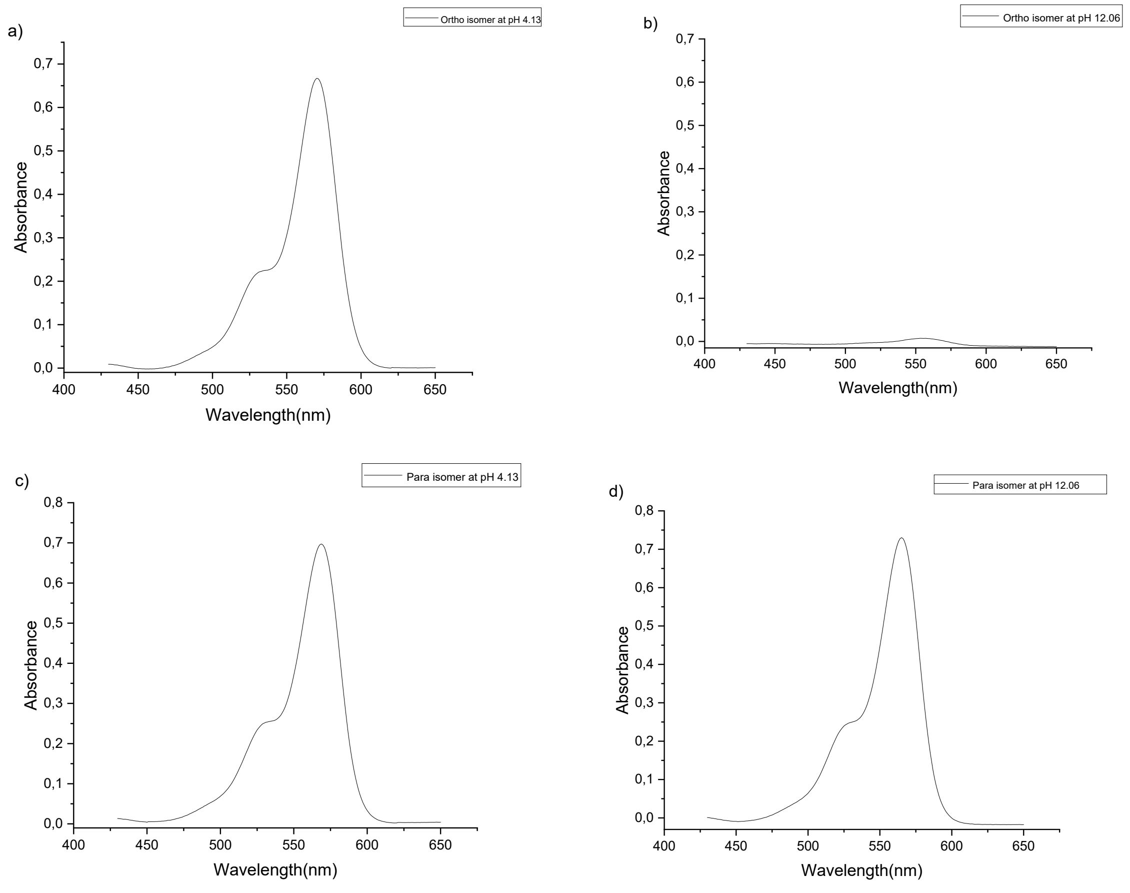

Figure S3. Absorption spectra of the obtained product with an R_f value of 0.30 (a,b) and of the product with an R_f value of 0.38 (c,d) at acidic (a,c) and basic pH (b,d). Measurements were performed in 25% aq. EtOH with HCl or NaOH to adjust the pH.

5-Sulfonamide isomer (R_f = 0.38): ^{1}H NMR (500 MHz, CD_3OD:$CDCl_3$ 1:1): δ (ppm) = 8.87 (d, J = 1.5 Hz, 1H, Ar-H), 8.48 (dd, J = 7.8 Hz, 1.5 Hz, 1H, Ar-H), 7.55 (d, J = 8.0 Hz, 1H, Ar-H), 7.34 (d, J = 9.5 Hz, 2H), 7.11 (dd, J = 9.5 Hz, 2.5 Hz, 2H, Ar-H), 7.00 (d, J = 2.0 Hz, 2H, Ar-H), 3.86 (s, 3H), 3.83 (m, 8H), 3.22 (m, 2H), 2.53 (m, 2H), 1.83 (m, 2H), 1.76 (m, 2H), 1.59 (m, 2H), 1.51 (m, 12H). ^{13}C NMR (125 MHz, CD_3OD:$CDCl_3$ 1:1): δ (ppm) = 178.6, 161.9, 160.9, 159.6, 150.3, 146.6, 137.7, 135.0, 136.5, 134.5, 131.7, 130.5, 118.0, 117.7, 99.7, 55.1, 49.6, 46.8, 37.6, 33.2, 29.9, 28.3, 16.0.

3-Sulfonamide isomer (R_f = 0.30): ^{1}H NMR (500 MHz, CD_3OD:$CDCl_3$ 1:1): δ (ppm) = 8.73 (d, J = 1.5 Hz, 1H, Ar-H), 8.42 (dd, J = 7.8 Hz, 1.65 Hz, 1H, Ar-H), 7.57 (d, J = 7.9 Hz, 1H, Ar-H), 7.32 (d, J = 9.5 Hz, 2H), 7.14 (dd, J = 9.6 Hz, 2.5 Hz, 2H, Ar-H), 7.03 (d, J = 2.4 Hz, 2H, Ar-H), 3.85 (m, 8H), 3.82 (s, 3H), 3.01 (m, 2H), 2.44 (m, 2H), 1.71 (m, 2H), 1.62 (m, 2H), 1.52 (m, 12H), 1.45 (m, 2H). ^{13}C NMR (125 MHz, CD_3OD:$CDCl_3$ 1:1): δ (ppm) = 178.4, 161.7, 159.7, 152.2, 145.0, 135.9, 135.8, 134.9, 133.4, 129.9, 117.9, 117.9, 99.9, 55.0, 49.7, 46.7, 37.4, 33.2, 29.7, 28.1, 16.0.

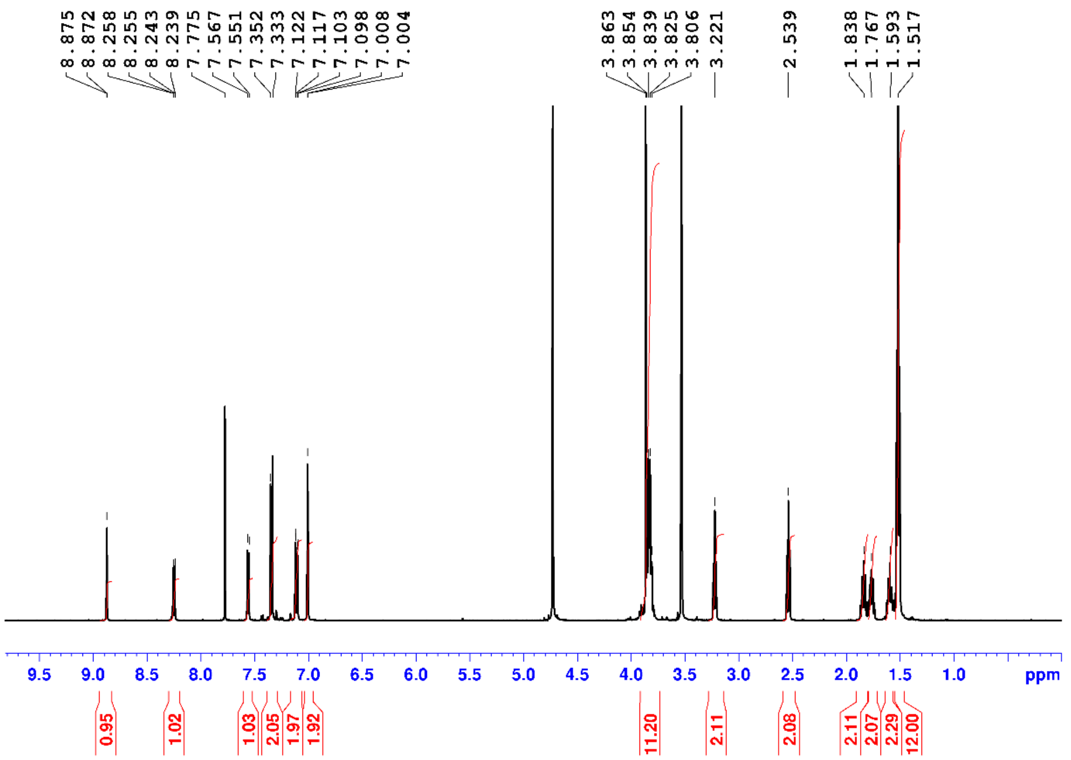

Figure S4. ^{1}H NMR of the 5-sulfonamide isomer (R_f = 0.38) in CD_3OD:$CDCl_3$ (1:1).

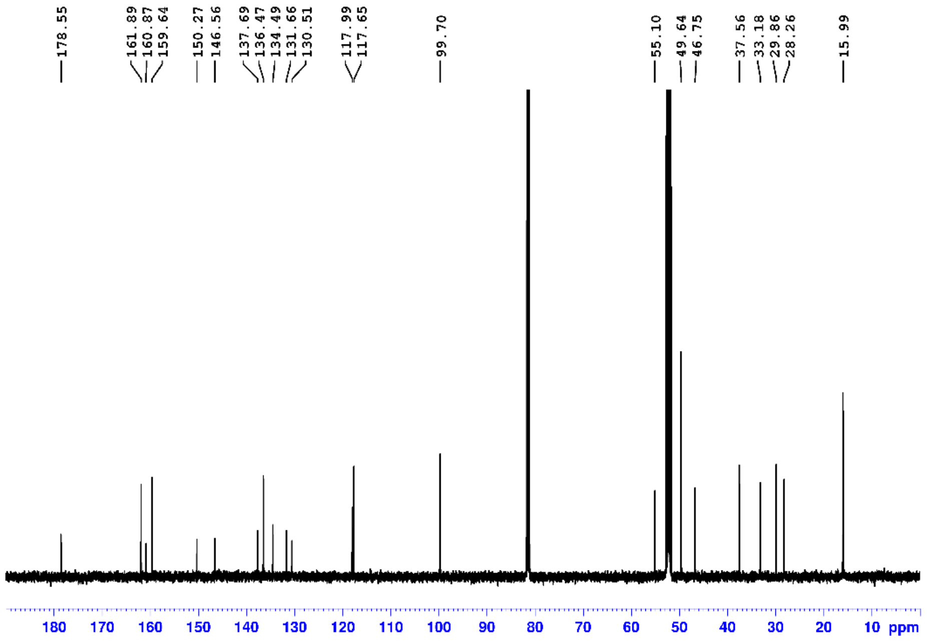

Figure S5. ^{13}C NMR of the 5-sulfonamide isomer (R_f = 0.38) in CD_3OD:$CDCl_3$ (1:1).

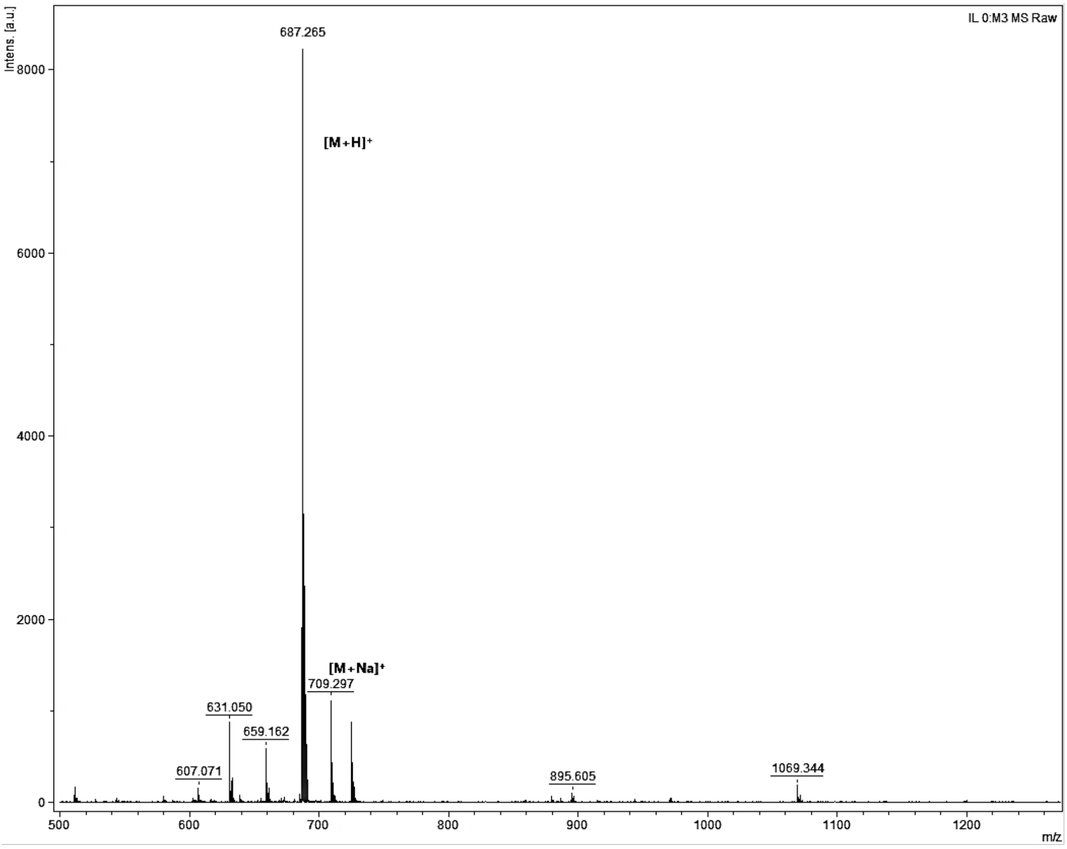

Figure S6. MALDI-MS of the 5-sulfonamide isomer (R_f = 0.38).

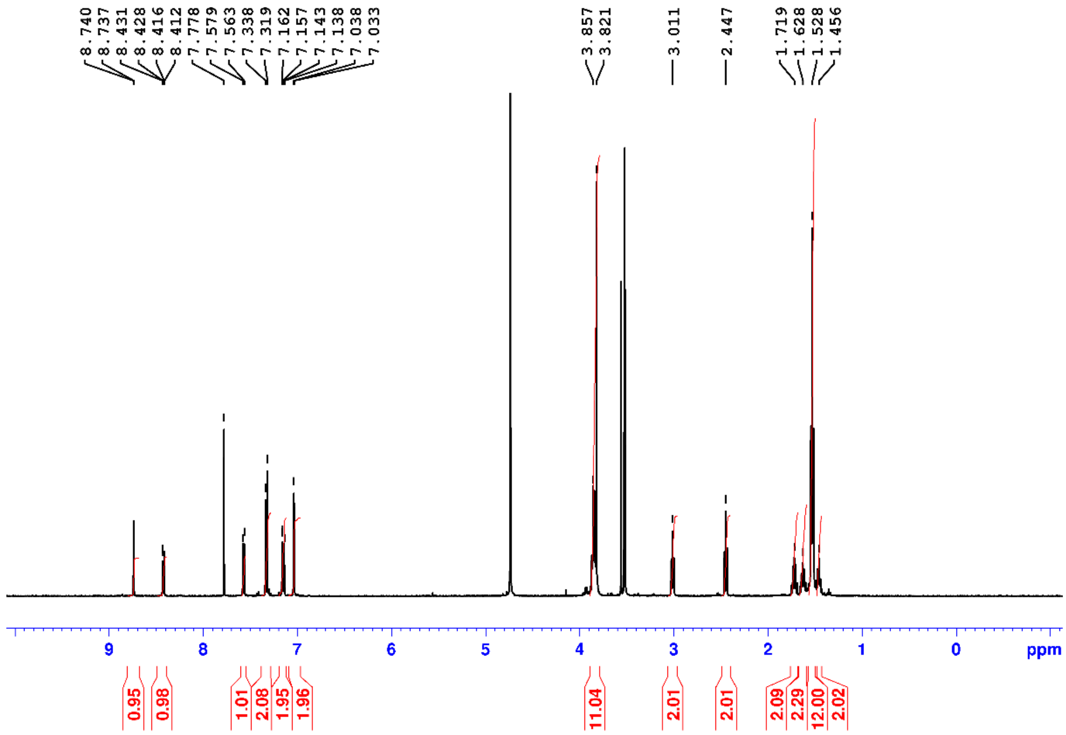

Figure S7. ^{1}H NMR of the 3-sulfonamide isomer (R_f = 0.30) in CD_3OD:$CDCl_3$ (1:1).

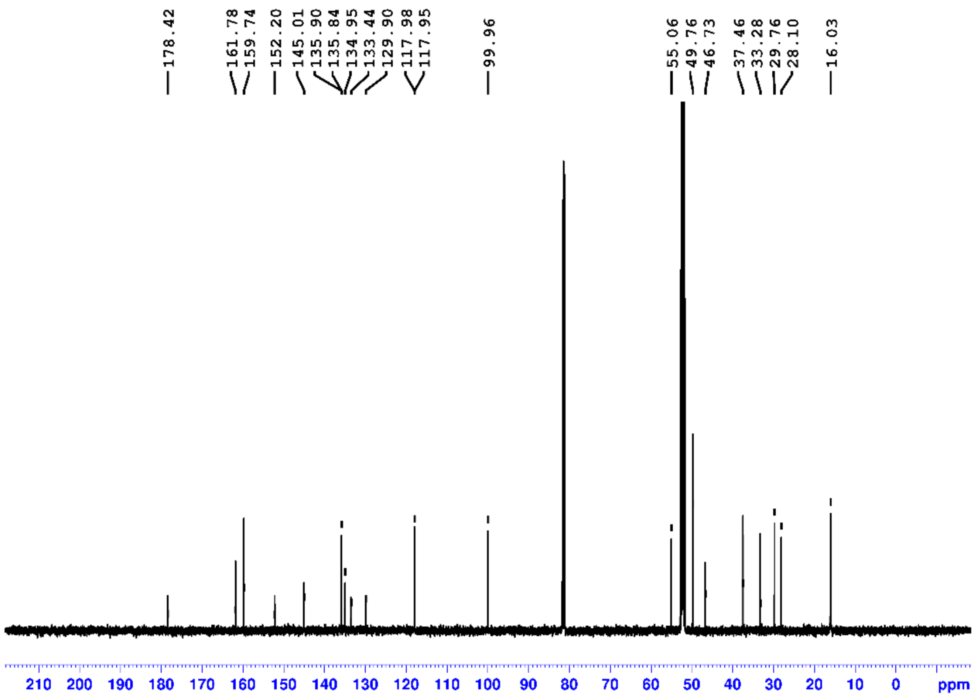

Figure S8. ^{13}C NMR of of the 3-sulfonamide isomer (R_f = 0.30) in CD_3OD:$CDCl_3$ (1:1).

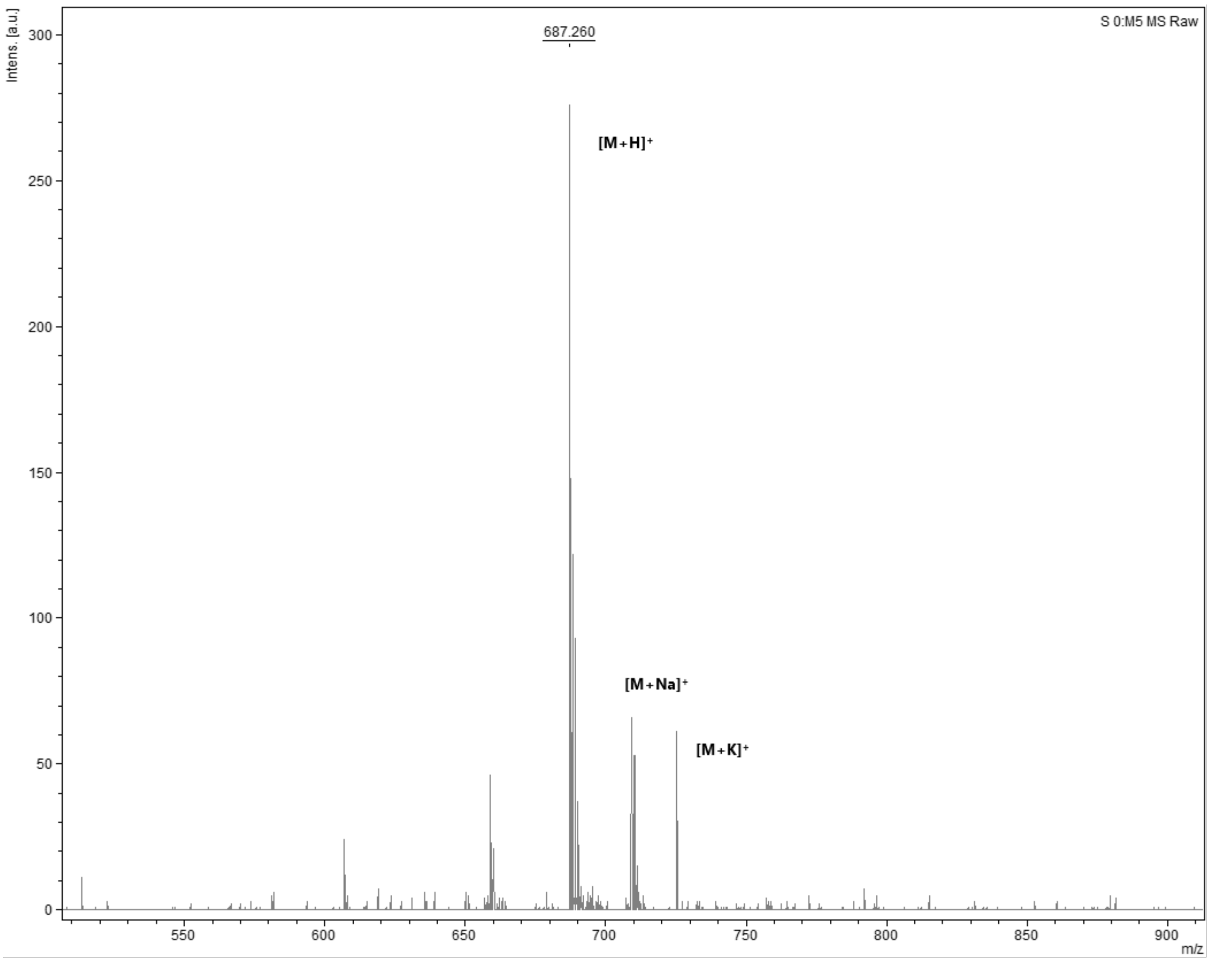

Figure S9. MALDI-MS of the 3-sulfonamide isomer (R_f = 0.30).

SRB-Ahx-OH. To a suspension of 0.12 g (0.25 mmol) of the para-isomer of SRB-Ahx-OMe in 10 mL of dioxane was added 10 mL of 6 M HCl dropwise over a period of 5 minutes. Then, the reaction mixture was stirred at room temperature for 20 hours and monitored by TLC. After that, the mixture is poured into 100 mL of water. The resulting solid was collected by filtration and purified by flash column chromatography using Büchi C 850 column prep with the silica 60 A (particle size 60-200 micron) and DCM/methanol (90:10) to afford 0.10 g (0.15 mol) SRB-Ahx-OH as a purple solid with a yield of 61%.

SRB-Ahx-OH: ^{1}H NMR (500 MHz, $CD_3OD:CDCl_3$ 1:1): δ (ppm) = 8.87 (d, J = 1.0 Hz, 1H, Ar-H), 8.25 (dd, J = 8.0 Hz, 1.5 Hz, 1H, Ar-H), 7.56 (d, J = 8.0 Hz, 1H, Ar-H), 7.33 (d, J = 9.5 Hz, 2H), 7.08 (dd, J = 9.5 Hz, 2.0 Hz, 2H, Ar-H), 7.00 (d, J = 2.0 Hz, 2H, Ar-H), 3.82 (m, 8H), 3.23 (m, 2H), 2.50 (m, 2H), 1.82 (m, 2H), 1.76 (m, 2H), 1.59 (m, 2H), 1.51(m, 12H). ^{13}C NMR (125 MHz, $CD_3OD:CDCl_3$ 1:1): δ (ppm) = 161.8, 159.6, 146.6, 136.3, 134.4, 131.6, 130.5, 117.9, 117.6, 99.6, 49.6, 46.7, 37.6, 33.1, 29.9, 28.2, 15.9.

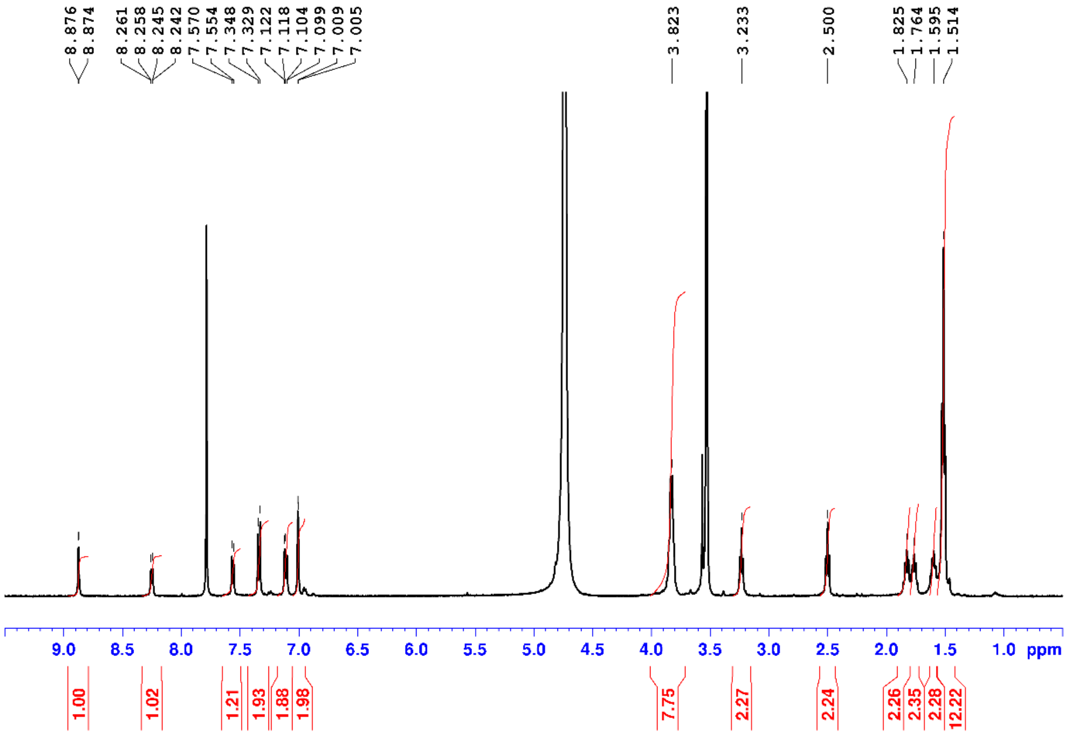

Figure S10. ^{1}H NMR of SRB-Ahx-OH in $CD_3OD:CDCl_3$ (1:1).

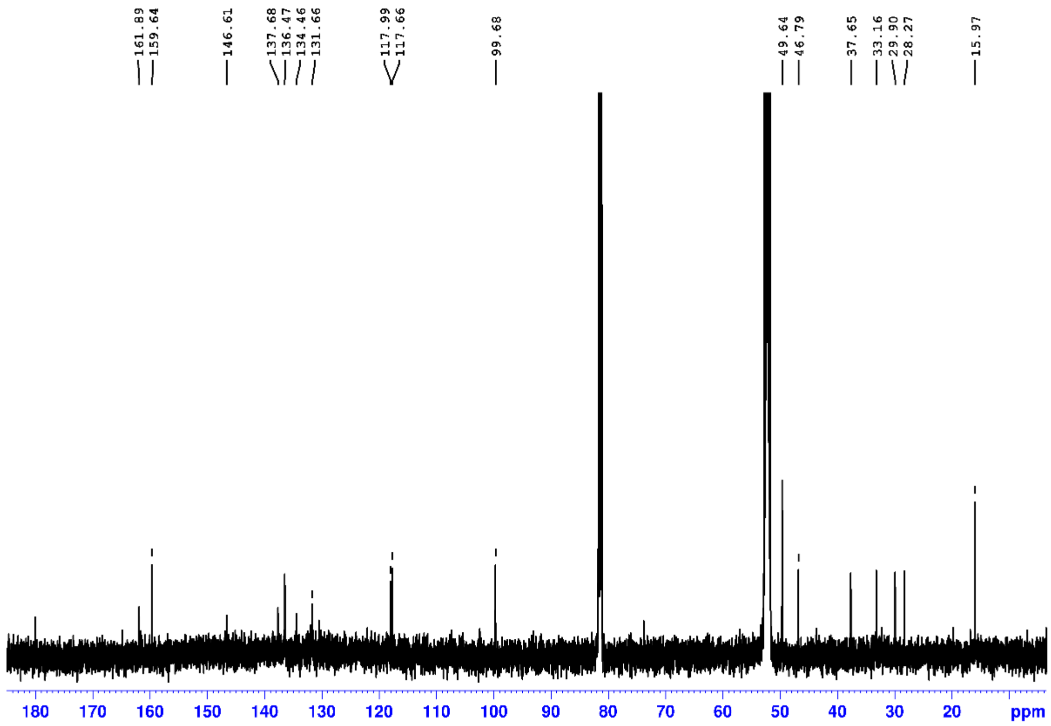

Figure S11. ^{13}C NMR of SRB-Ahx-OH in CD_3OD:$CDCl_3$ (1:1).

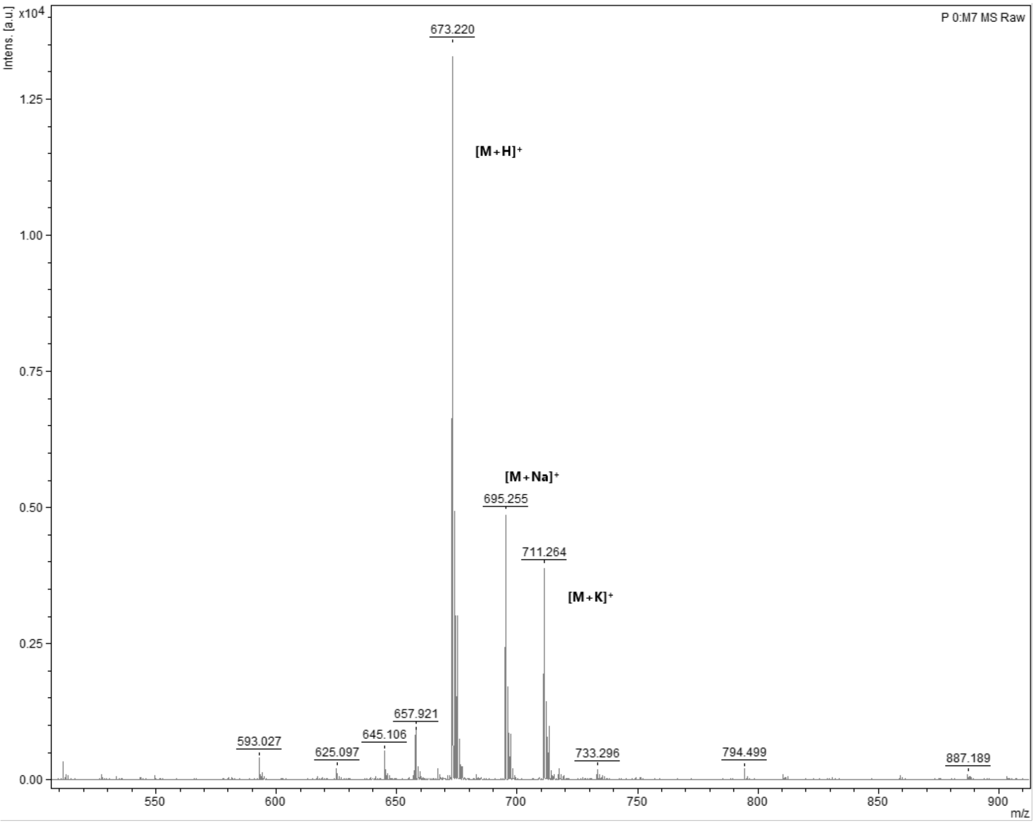

Figure S12. MALDI of SRB-Ahx-OH.

Oligoarginine Peptide Synthesis

Peptides were synthesized using Fmoc-based standard solid-phase peptide synthesis (SPPS) protocols.[3-4] Rink amide resin was used to obtain C-terminally amidated peptides and Fmoc-Arg(Pbf)-Wang resin was used for peptides with a carboxy terminus. For the side chain of arginine, the 2,2,4,6,7-pentamethyldihydrobenzofuran-5-sulfonyl group (Pbf) was used as a protecting group, which belongs to most easily removable groups during final cleavage with TFA.[5]

In a typical procedure, 0.1 mmol of the resin was loaded into a reactor for peptide synthesis (10 ml, pore size 25 µm from Carl Roth GmbH & Co. KG) and swollen in 5 ml DMF for 1 hour. After washing with 3 × 4 ml DMF, the Fmoc protecting group on the Fmoc-Arg(Pbf)-Wang resin or Rink amide resin was removed using 2 ml 20% (v/v) piperidine in DMF (2 times) for 20 min (each time 10 min). The resin was washed thoroughly three times with DMF and the deprotection was confirmed by the Kaiser test with blue color.[6]

For coupling and deprotection cycles, 4 eq (0.4 mmol) of the Fmoc-protected amino acid (e.g. Fmoc-Arg(Pbf)-OH) and 0.4 mmol HCTU were combined as solids, 5 ml of 0.117 M DIPEA in DMF was added and the mixture was allowed to react for 5 min. Then, the mixture was added to the resin and mildly shaken for 30 min. Finally, the solution was drained and then washed with 3 × 4 ml DMF. Complete coupling was confirmed with a colorless solution in the Kaiser test.[6] Deprotection was then performed with 2 ml 20% piperidine in DMF (2 times) for 20 min (each time 10 min) and the resin was washed thoroughly three times with DMF.

For the final cleavage of the peptide, 5 ml of a freshly prepared cleavage cocktail with a mixture of 90% TFA, 5% thioanisole, 3% 1,2-ethanedithiol, and 2% anisole was used.[7] The resin-bound peptide was added to the cleavage cocktail and mildly shaken for 3 hours. Then, the product was recovered by precipitation in cold diethyl ether, followed by centrifugation to separate the filtrate from the residue. The product was lyophilized overnight and the dried product was characterized by MALDI and purified by RP-HPLC to >95% purity.

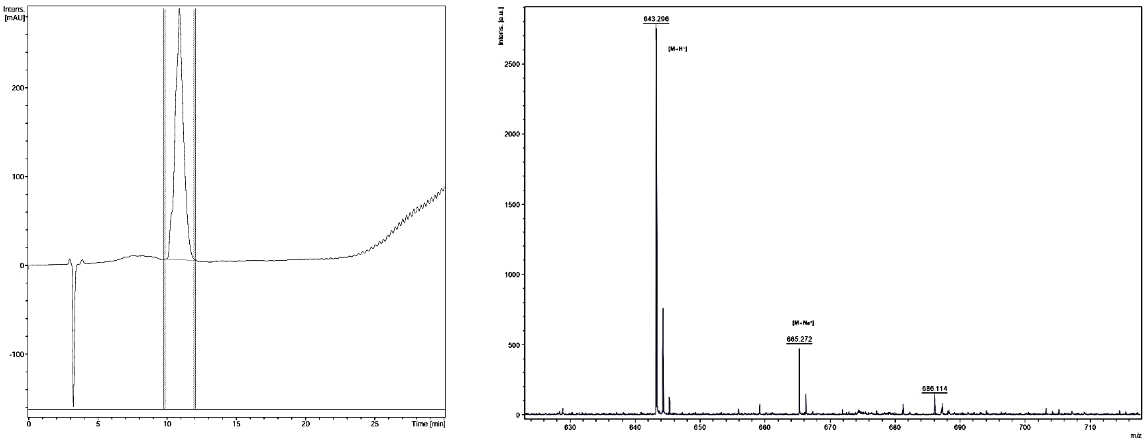

Figure S13. HPLC trace and MALDI of H-(Arg)$_4$-OH.

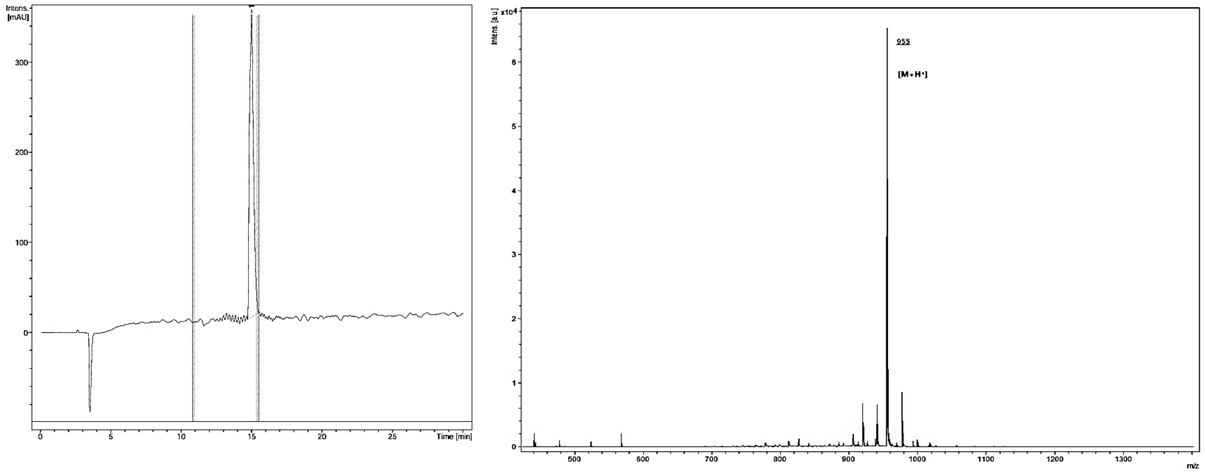

Figure S14. HPLC trace and MALDI of H-(Arg)$_6$-OH.

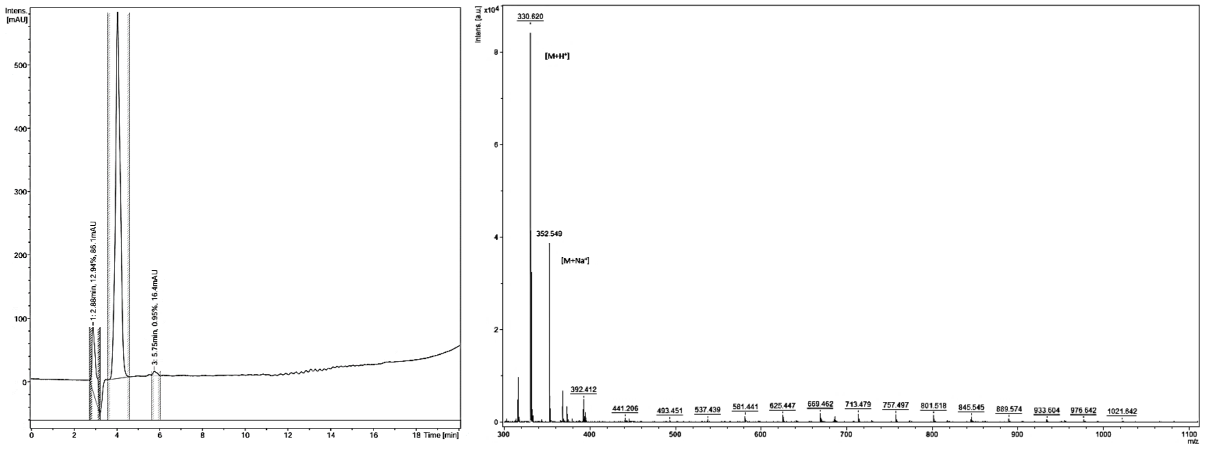

Figure S15. HPLC trace and MALDI of H-(Arg)$_2$-NH$_2$.

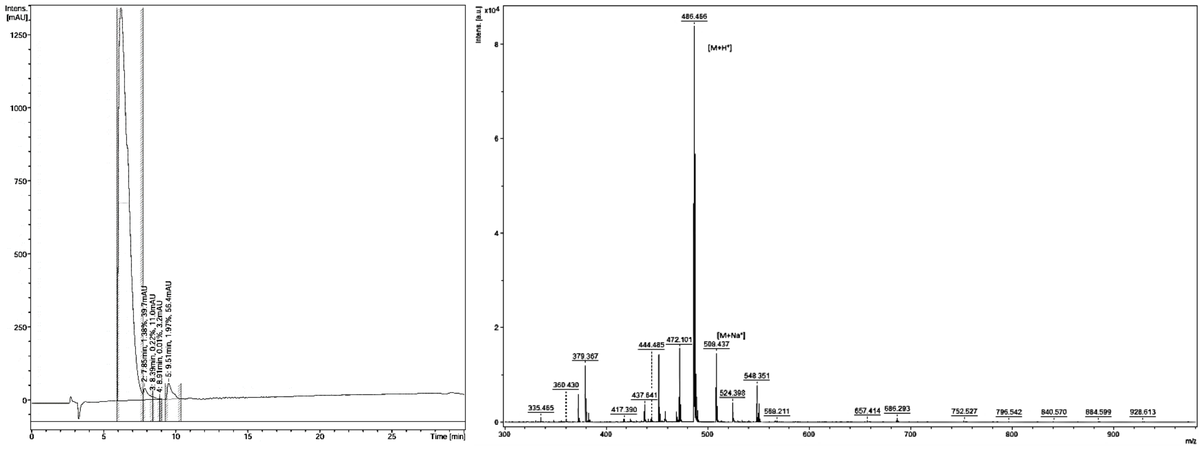

Figure S16. HPLC trace and MALDI of H-(Arg)$_3$-NH$_2$.

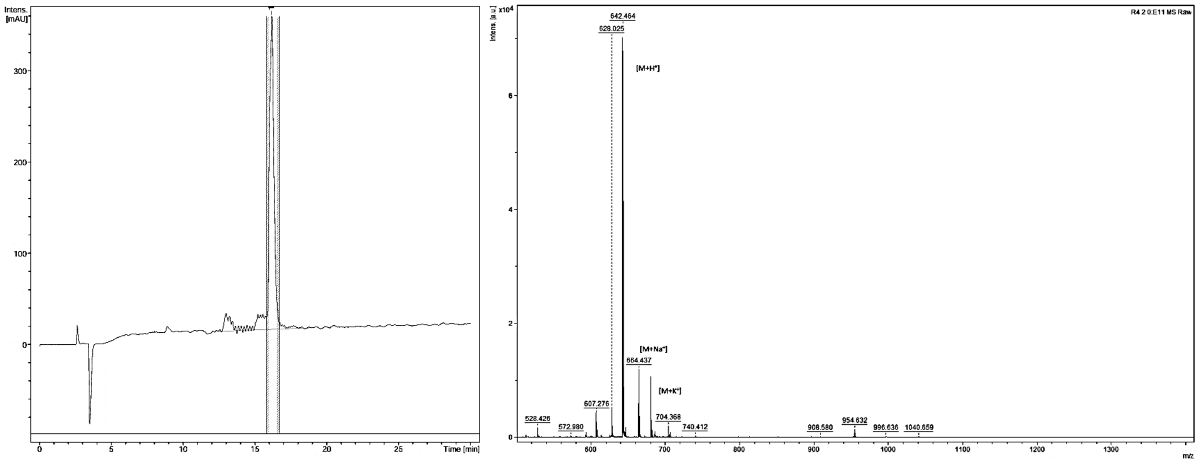

Figure S17. HPLC trace and MALDI of H-(Arg)$_4$-NH$_2$.

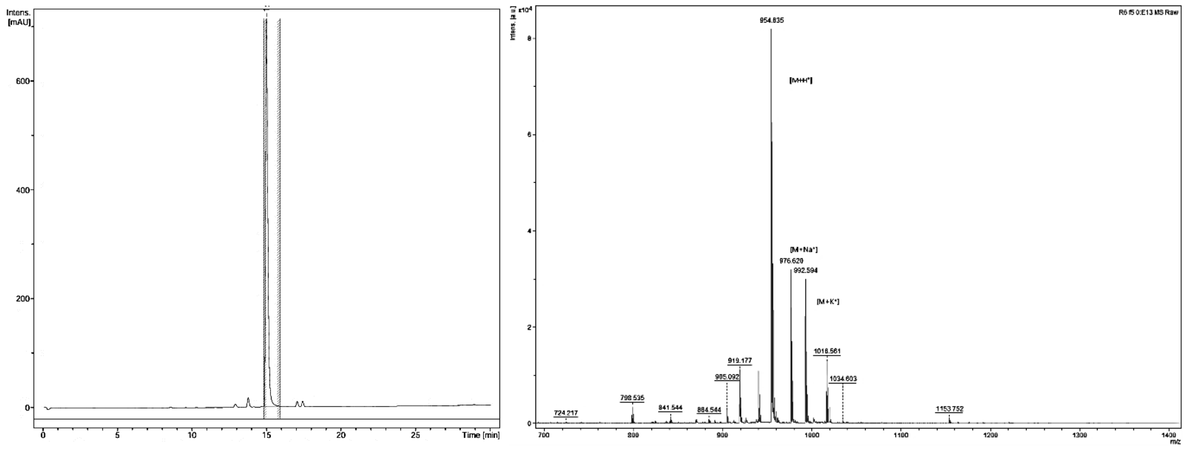

Figure S18. HPLC trace and MALDI of H-(Arg)$_6$-NH$_2$.

Synthesis of Fluorescently Labelled Peptides

SRB-Ahx-(Arg)$_4$-OH: The peptide sequence SRB-Ahx-(Arg)$_4$-OH was synthesized manually by SPPS. After the Fmoc-Arg(Pbf)-Wang resin (0.05 mmol) was swollen for 1 h in 2.5 ml DMF, the Fmoc-protecting group was removed by 1 ml of 20% piperidine in DMF (2 times) for 20 minutes (each time 10 minutes). Then, the Fmoc-protected amino acids were sequentially coupled to the resin. Therefore, each amino acid was activated by combining the Fmoc-protected amino acid (4 eq, 0.2 mmol) and HCTU (4 eq, 0.2 mmol) as solids, addition of 2.5 ml of 0.117 M DIPEA, and 5 min with mild shaking. Then, the mixture was added to the resin and mildly shaken for 30 min before the solution was drained and washed with 3 × 4 ml DMF. Complete coupling was confirmed with a colorless solution in the Kaiser test,[6] and deprotection was then performed with 2 ml 20% piperidine in DMF (2 times) for 20 min (each time 10 min). Finally, the resin was washed thoroughly three times with DMF.

For the last step, 2 eq (0.1 mmol) SRB-Ahx-OH was mixed 4 eq (0.2 mmol) HCTU and activated in 2 ml of 0.117 M DIPEA in DMF and for 5 min. The mixture was added to the resin and allowed to react for overnight. After coupling, excess dye was removed by washing the resin properly with copious amounts of DMF until the drained solution became colorless.

The peptide was cleaved from the resin using 2.5 ml of a freshly prepared cleavage cocktail with a mixture of 90% TFA, 5% thioanisole, 3% 1,2-ethanedithiol, and 2% anisole for 3 h. Then, the product was recovered by precipitation in cold diethyl ether, followed by centrifugation to separate the filtrate from the residue. After that the product was lyophilized overnight. The dried product was characterized by MALDI (**Figure S19**) and purified by RP-HPLC to obtain the peptide with a purified yield of 34% (**Figure S20**).

FL-Ahx-(Arg)$_8$-NH$_2$: The FITC-labelled peptide was synthesized using 0.1 mmol of the Rink amide resin as described above to obtain a H-(Arg)$_8$-Rink amide resin. Then, 2 eq (0.2 mmol) Fmoc-Ahx-OH, pre-activated with 0.2 mmol HCTU in 5 ml of 0.117 M DIPEA in DMF was added and reacted for 30 min. After deprotection, 2 eq FITC (0.2 mmol) in 2 ml DMF was added and reacted for overnight. Then, to remove the excess of dye, it was washed properly with excess of DMF until the drained solution becomes colorless. The peptide was cleaved from the resin using a cleavage cocktail of 5 ml 5 ml of a freshly prepared cleavage cocktail with a mixture of 90% TFA, 5% thioanisole, 3% 1,2-ethanedithiol, and 2% anisole for 3 h. The product was recovered by precipitating the filtrate in cold diethyl ether, followed by centrifugation. After that the product was lyophilized for overnight. The dried product was characterized by MALDI (**Figure S21**) and purified by RP-HPLC with a yield of 40% (**Figure S22**).

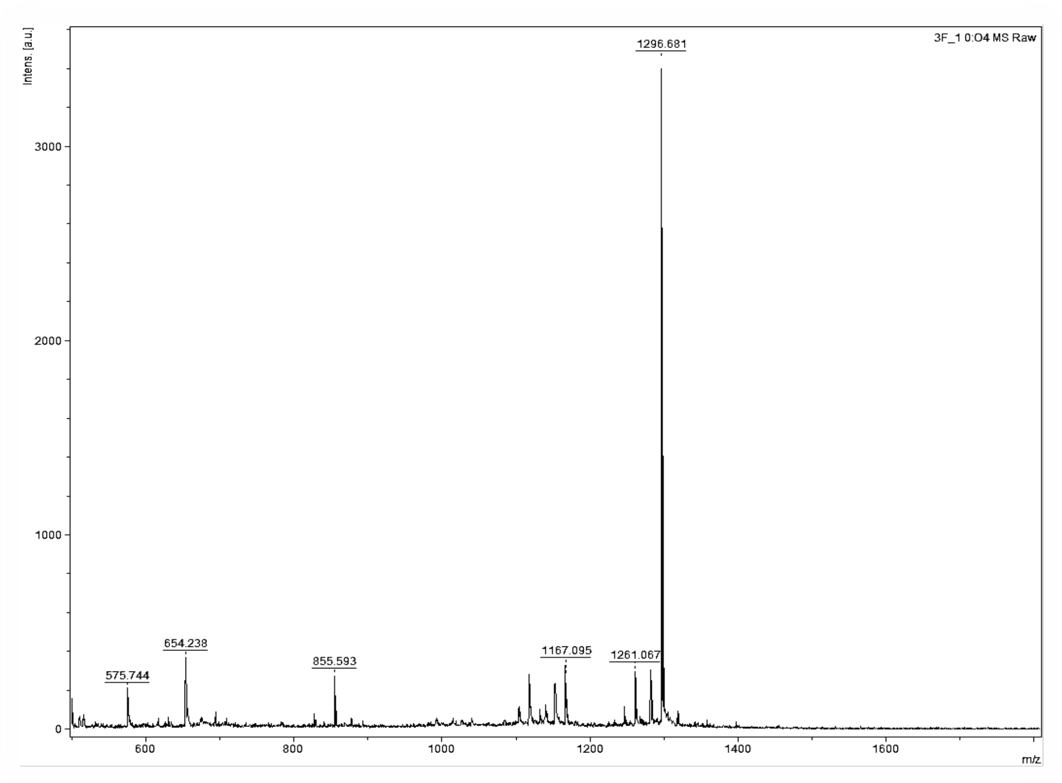

Figure S19. MALDI of SRB-Ahx-$(Arg)_4$-OH.

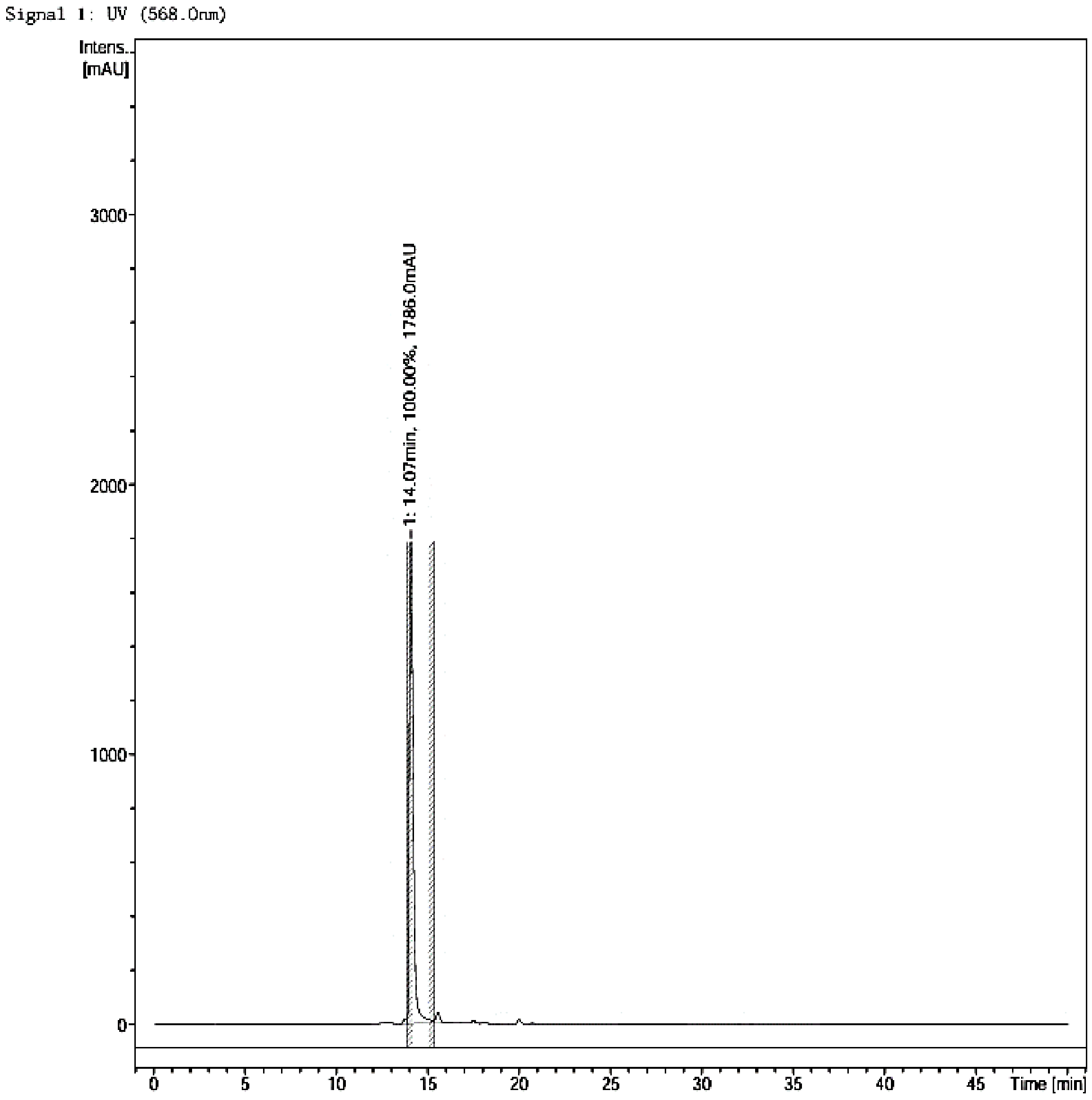

Figure S20. HPLC trace of SRB-Ahx-(Arg)$_4$-OH.

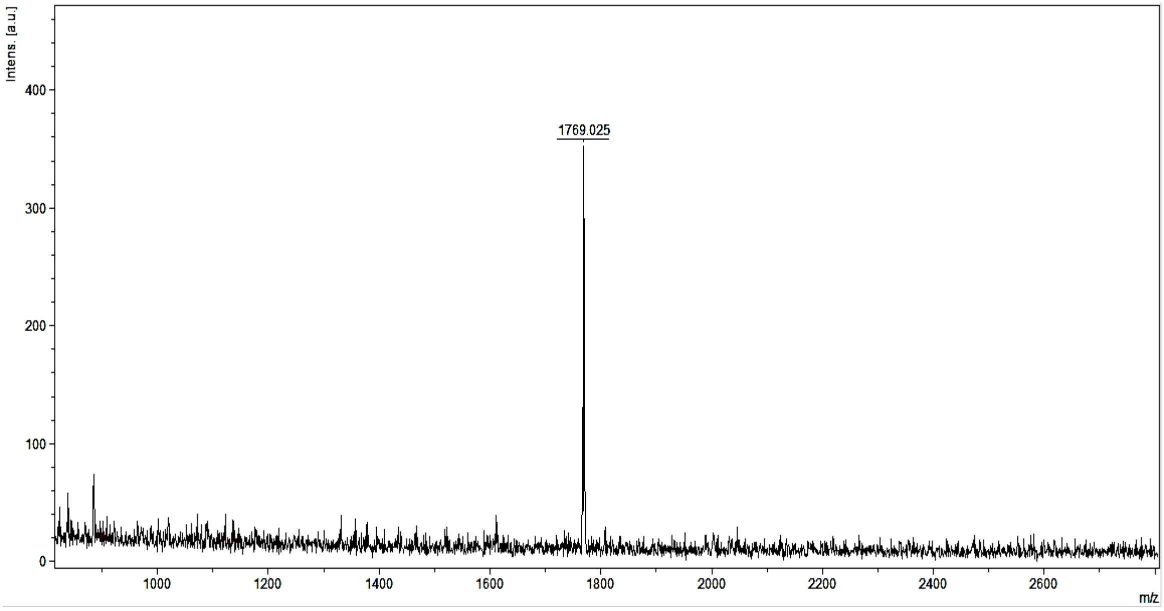

Figure S21. MALDI of FL-Ahx-$(Arg)_8$-NH_2.

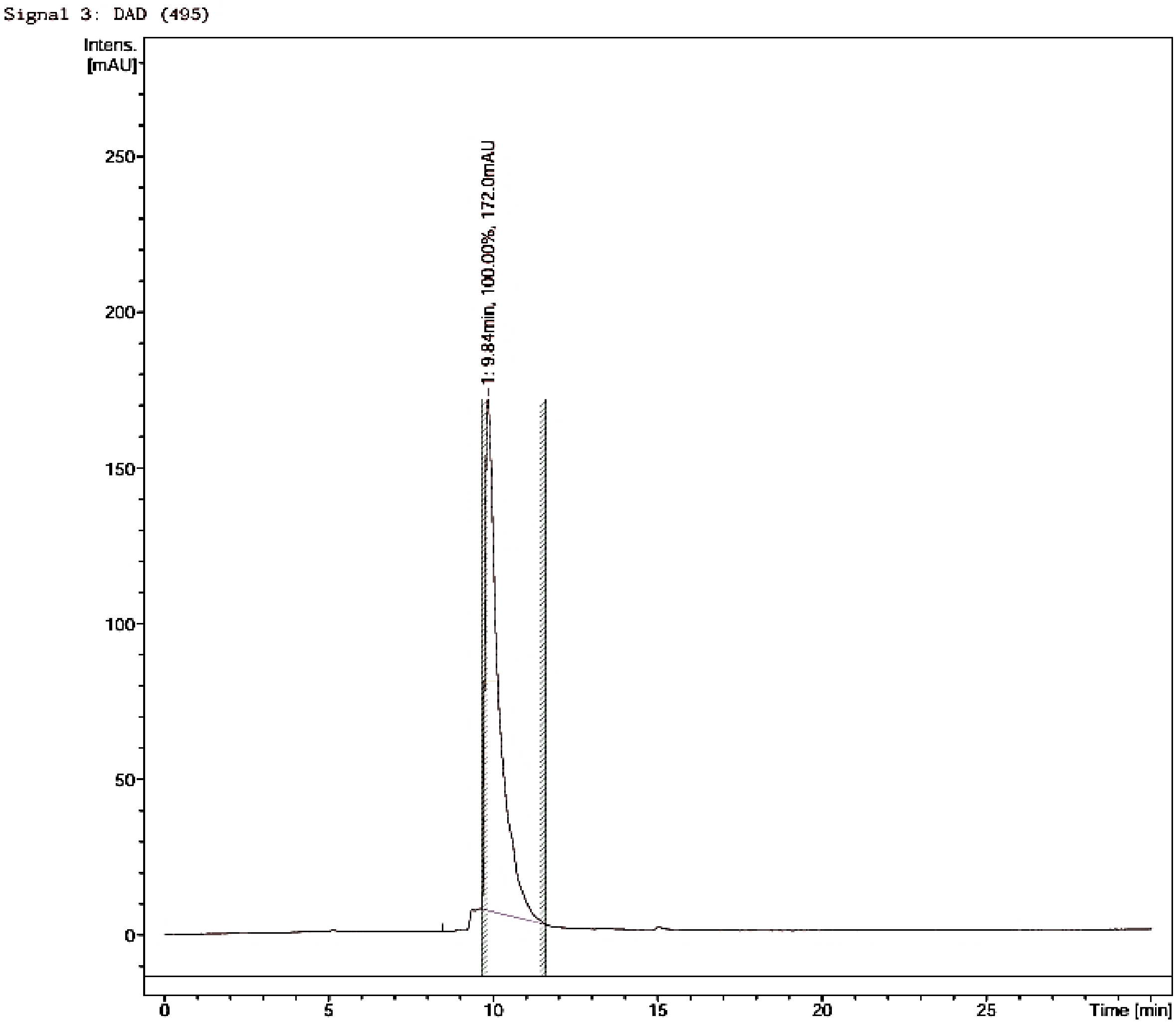

Figure S22. HPLC trace of FL-Ahx-$(Arg)_8$-NH_2.

Determination of Binding Constants

Determination of Peptide Concentrations

The peptide concentration was determined by measuring their absorbance at 214 nm and considering their respective extinction coefficients.[8] For the molecular weight of the peptides, the fully protonated trifluoroacetate salt was used.

**Peptides**	ε_{214} (M^{-1} cm^{-1})		
	Peptide bonds	Arg side chains	Σ
**H-(Arg)$_2$-OH**	1×923	2×102	1127
**H-(Arg)$_3$-OH**	2×923	3×102	2148
**H-(Arg)$_4$ -OH**	3×923	4×102	3177
**H-(Arg)$_6$ -OH**	5×923	6×102	5227
**H-(Arg)$_2$-NH$_2$**	2×923	2×102	2050
**H-(Arg)$_3$ -NH$_2$**	3×923	3×102	3075
**H-(Arg)$_4$ -NH$_2$**	4×923	4×102	4100
**H-(Arg)$_6$ -NH$_2$**	6×923	6×102	6150

For SRB-Ahx-(Arg)$_4$-OH, a molar extinction coefficient of ε = 90,800 M^{-1} cm^{-1} at λ = 572 nm was used,[9] and for FL-Ahx-(Arg)$_8$-NH$_2$, a molar extinction coefficient of ε = 77,000 M^{-1} cm^{-1} at λ= 494 nm was used.[10]

Competitive Titrations with Oligoarginine Peptides

Competitive fluorescence titrations were performed as previously described[11-13] and the resulting titration curves were analyzed using established procedures. Competitive titrations were either analyzed with a fitting function assuming 1:1 host-competitor complexes[12] or with a fitting function involving higher complexes with more than one host bound per peptide.[11, 13] In the latter case, an identical binding affinity is assumed for all binding sites. The reported errors are standard errors obtained from the fitting functions with 95% confidence intervals.

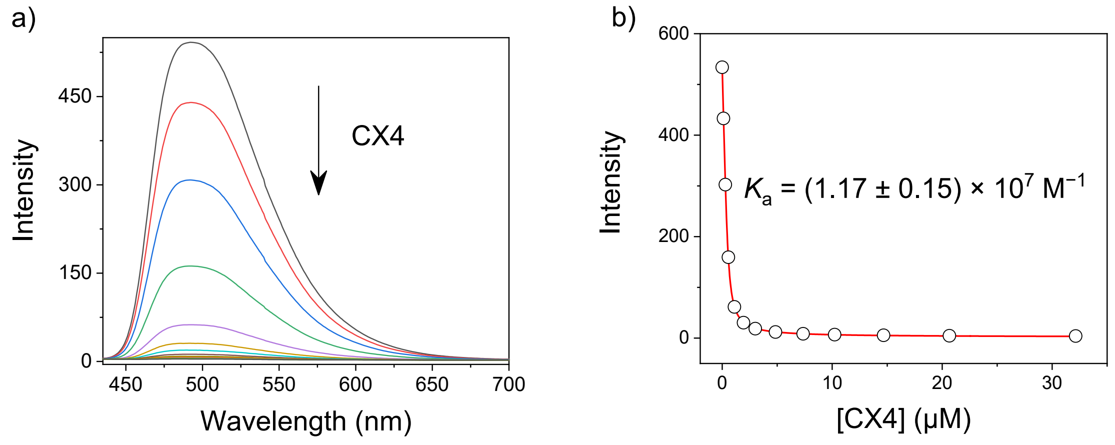

Figure S23. a) Fluorescence titration of 0.5 µM LCG (λ_{ex} = 369 nm) with varying concentrations of CX4 in 10 mM mM NaH$_2$PO$_4$, pH 7.2, 25 °C. b) Respective titration curve (λ_{em} = 502 nm) with fitted line.

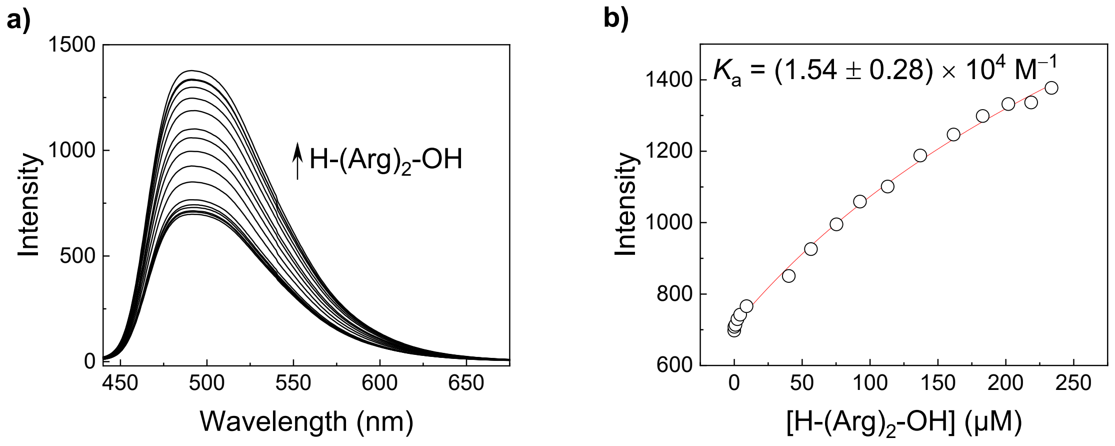

Figure S24. a) Competitive fluorescence titration with 0.5 μM LCG, 1 μM CX4 (λ_{ex} = 369 nm) and varying concentrations of H-$(Arg)_2$-OH in 10 mM NaH_2PO_4, pH 7.2, 25 °C. b) Respective titration curve (λ_{em} = 492 nm) with fitted line.

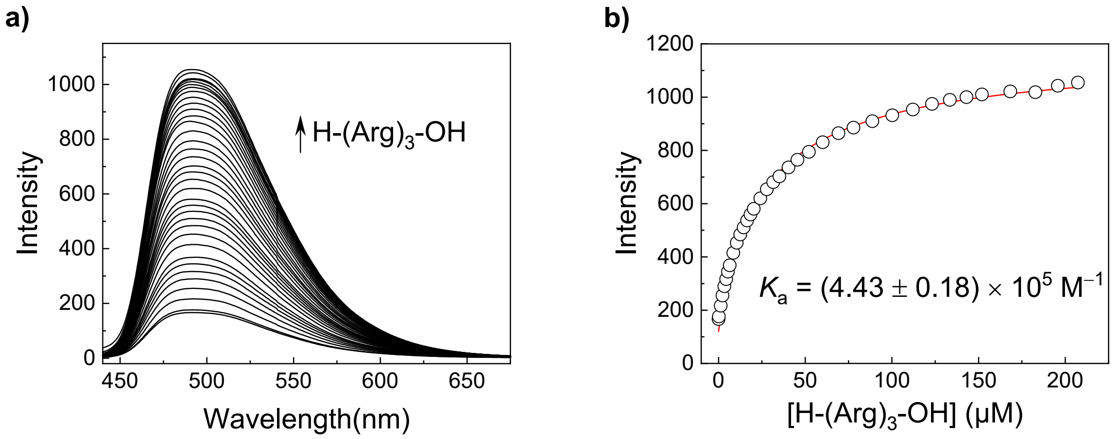

Figure S25. a) Competitive fluorescence titration with 0.5 μM LCG, 1 μM CX4 (λ_{ex} = 369 nm) and varying concentrations of H-$(Arg)_3$-OH in 10 mM NaH_2PO_4, pH 7.2, 25 °C. b) Respective titration curve (λ_{em} = 492 nm) with fitted line.

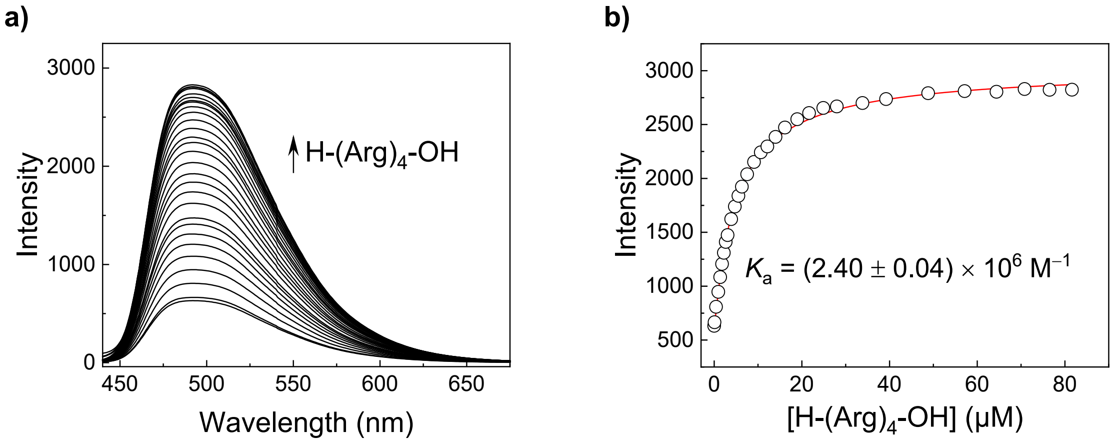

Figure S26. a) Competitive fluorescence titration with 0.5 μM LCG, 1 μM CX4 (λ_{ex} = 369 nm) and varying concentrations of H-$(Arg)_4$-OH in 10 mM NaH_2PO_4, pH 7.2, 25 °C. b) Respective titration curve (λ_{em} = 492 nm) with fitted line.

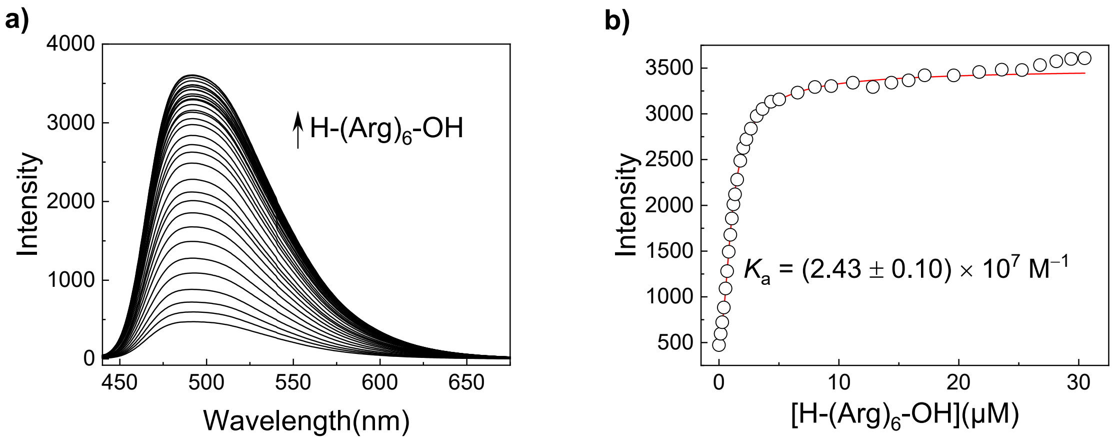

Figure S27. a) Competitive fluorescence titration with 0.5 μM LCG, 1 μM CX4 (λ_{ex} = 369 nm) and varying concentrations of H-(Arg)$_6$-OH in 10 mM NaH_2PO_4, pH 7.2, 25 °C. b) Respective titration curve (λ_{em} = 492 nm) with fitted line.

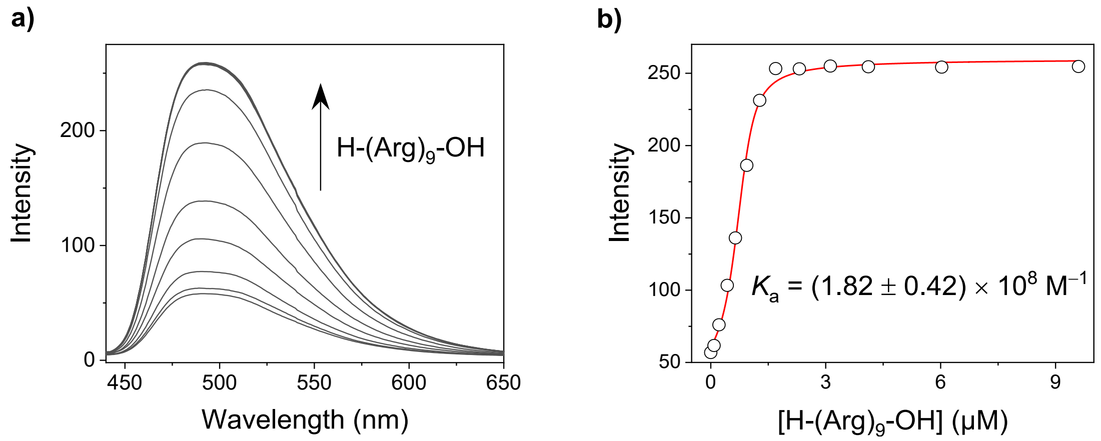

Figure S28. a) Competitive fluorescence titration with 0.5 μM LCG, 1 μM CX4 (λ_{ex} = 369 nm) and varying concentrations of H-(Arg)$_9$-OH in 10 mM NaH_2PO_4, pH 7.2, 25 °C. b) Respective titration curve (λ_{em} = 502 nm) with fitted line.

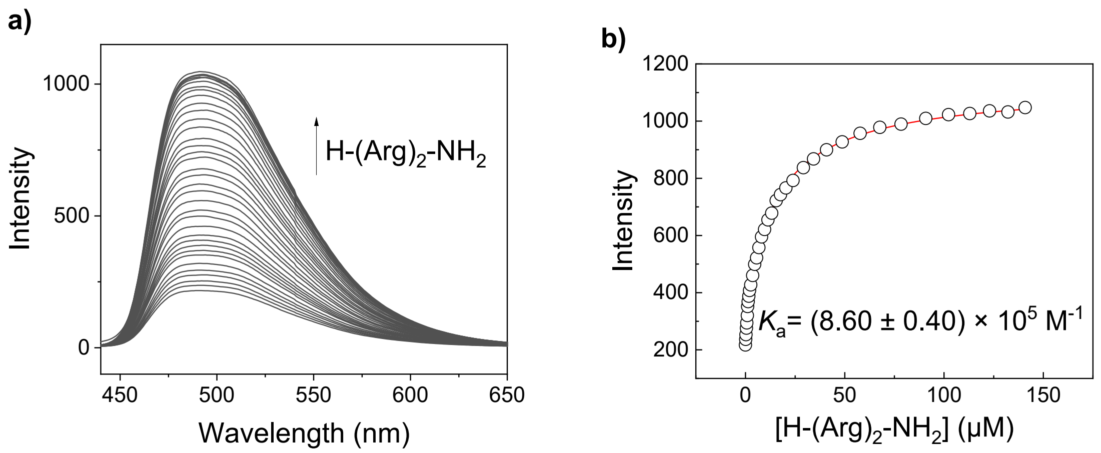

Figure S29. a) Competitive fluorescence titration with 0.5 μM LCG, 1 μM CX4 (λ_{ex} = 369 nm) and varying concentrations of H-(Arg)$_2$-NH_2 in 10 mM NaH_2PO_4, pH 7.2, 25 °C. b) Respective titration curve (λ_{em} = 492 nm) with fitted line.

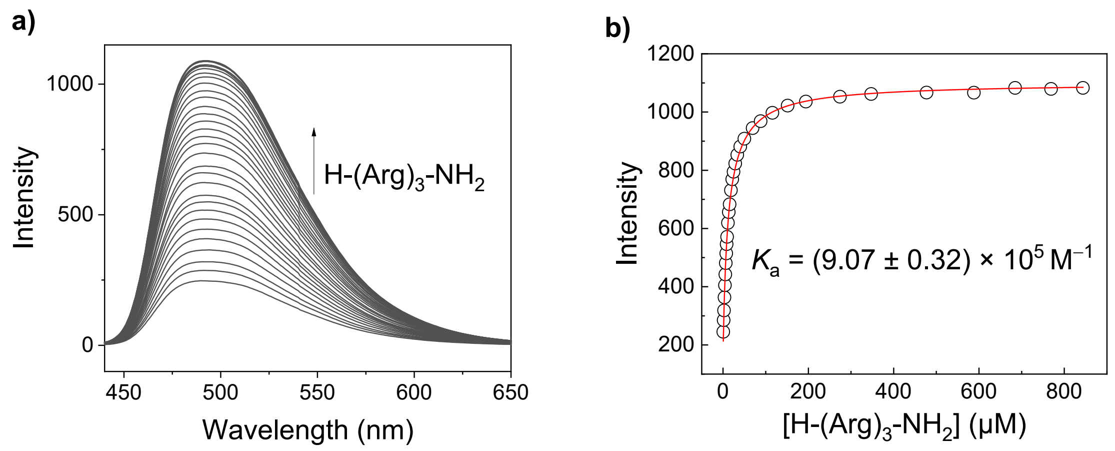

Figure S30. a) Competitive fluorescence titration with 0.5 µM LCG, 1 µM CX4 (λ_{ex} = 369 nm) and varying concentrations of H-(Arg)$_3$-NH$_2$ in 10 mM NaH$_2$PO$_4$, pH 7.2, 25 °C. b) Respective titration curve (λ_{em} = 492 nm) with fitted line.

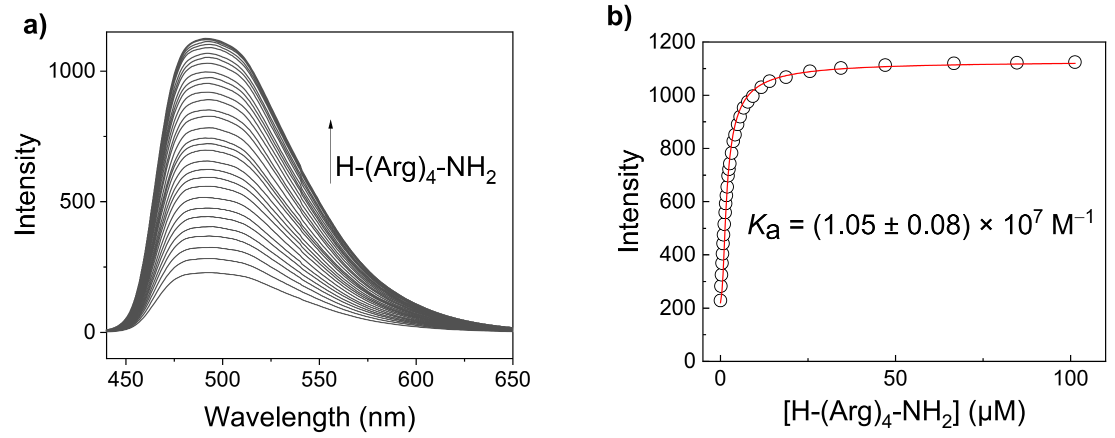

Figure S31. a) Competitive fluorescence titration with 0.5 µM LCG, 1 µM CX4 (λ_{ex} = 369 nm) and varying concentrations of H-(Arg)$_4$-NH$_2$ in 10 mM NaH$_2$PO$_4$, pH 7.2, 25 °C. b) Respective titration curve (λ_{em} = 492 nm) with fitted line.

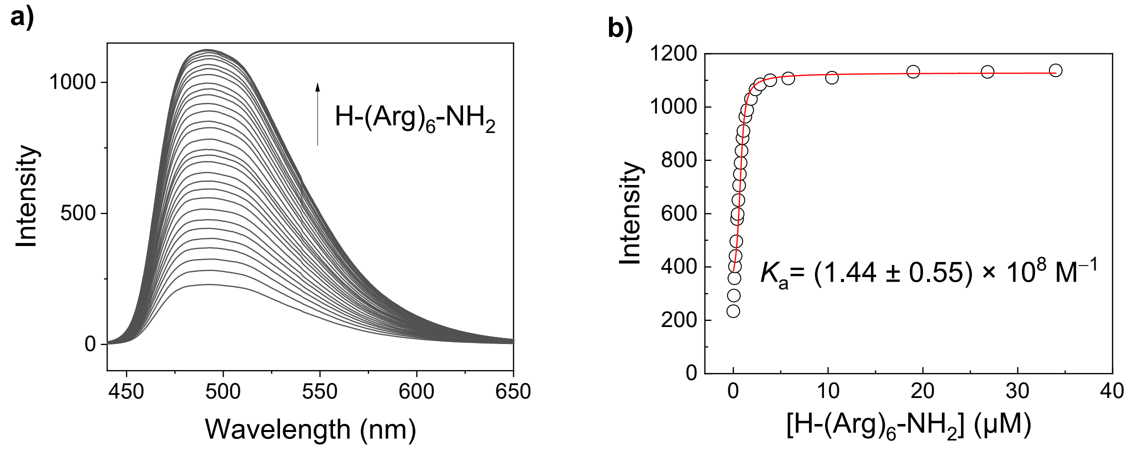

Figure S32. a) Competitive fluorescence titration with 0.5 µM LCG, 1 µM CX4 (λ_{ex} = 369 nm) and varying concentrations of H-(Arg)$_6$-NH$_2$ in 10 mM NaH$_2$PO$_4$, pH 7.2, 25 °C. b) Respective titration curve (λ_{em} = 492 nm) with fitted line.

Isothermal Titration Calorimetry (ITC)

ITC experiments were carried out at 25 °C on a VP-ITC from Microcal Inc. (Northampton, MA, United States). The solutions were degassed and thermostatted by a ThermoVac accessory. The titrations were performed by a series of injections of a highly concentrated compound in the injection syringe into the ITC cell containing a solution with low concentration of the other compound in 10 mM NaH_2PO_4, pH 7.2. The same buffer was also used in the reference cell. The data was analyzed with the Origin 7.0 software provided by the instrument manufacturer to obtain the binding constants. The reported errors of the binding constants are standard errors obtained from the fitting functions with 95% confidence intervals. Dilution heats were not accounted for as these were small (<10% of the overall heat, see Fig. S34) and thus have a negligible influence on the binding constants.

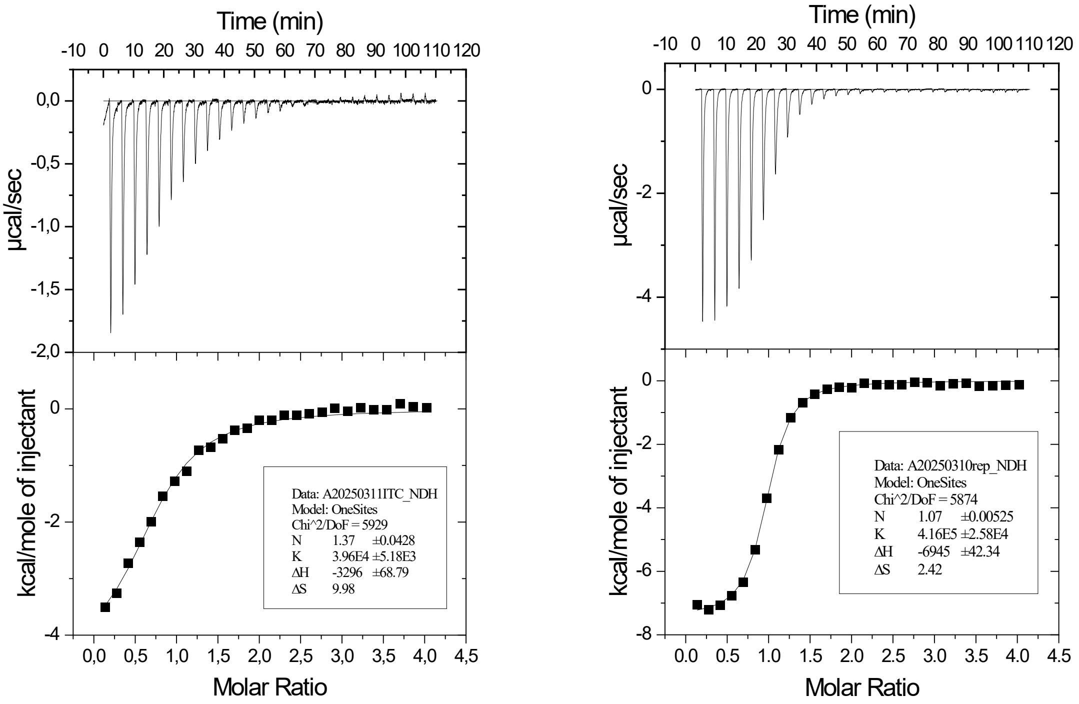

Figure S33. ITC direct binding titration of 2 mM H-$(Arg)_2$-OH (left) and 2 mM H-$(Arg)_3$-OH (right) into 100 µM CX4 in 10 mM NaH_2PO_4, pH 7.2, 25 °C.

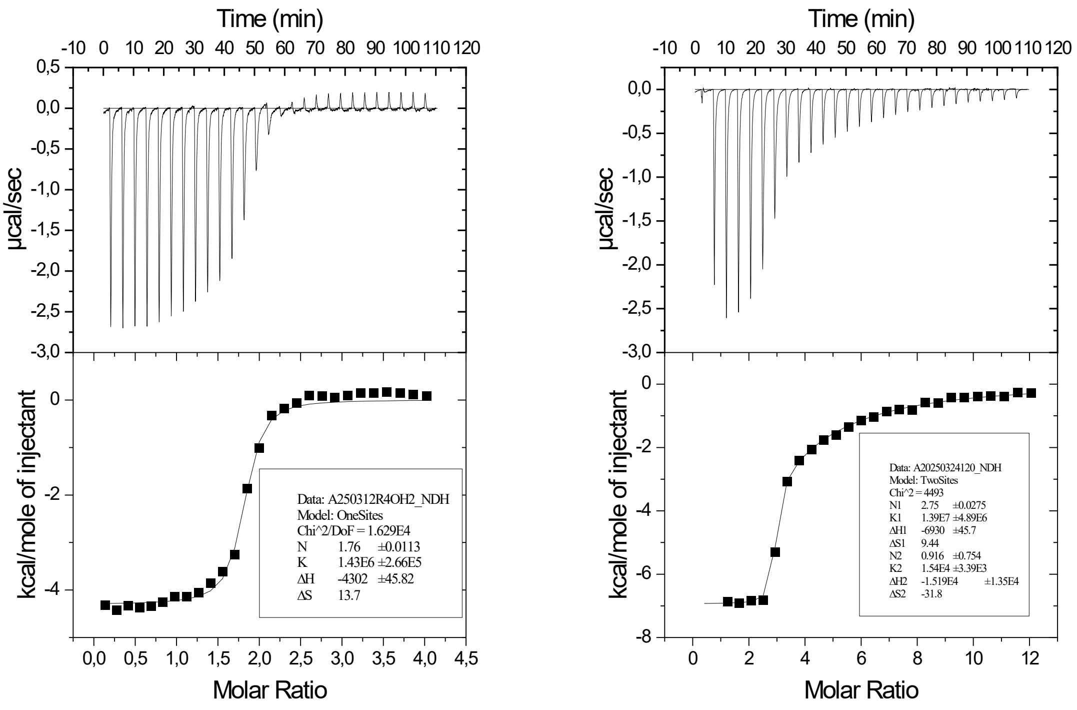

Figure S34. ITC direct binding titration of 2 mM H-(Arg)$_4$-OH into 100 µM CX4 (left) and 1.2 mM CX4 into 20 µM H-(Arg)$_6$-OH in 10 mM NaH_2PO_4, pH 7.2, 25 °C.

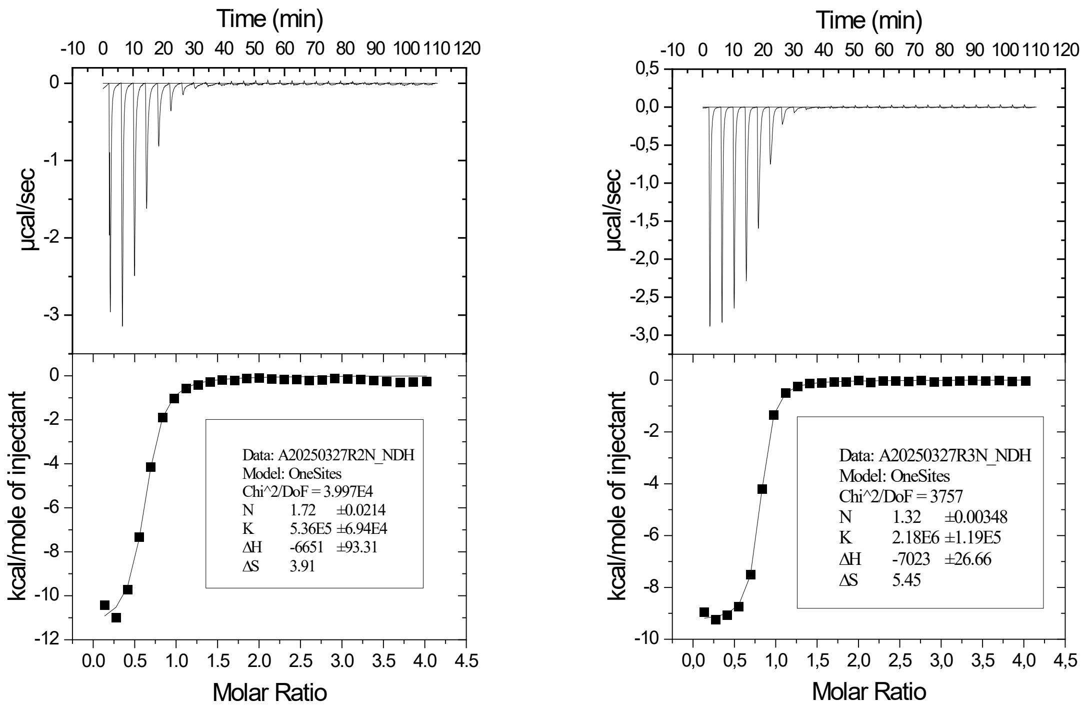

Figure S35. ITC direct binding titration of 2 mM H-(Arg)$_2$-NH$_2$ (left) and 2 mM H-(Arg)$_3$-NH$_2$ (right) into CX4 (100 µM) in 10 mM NaH_2PO_4, pH 7.2, 25 °C.

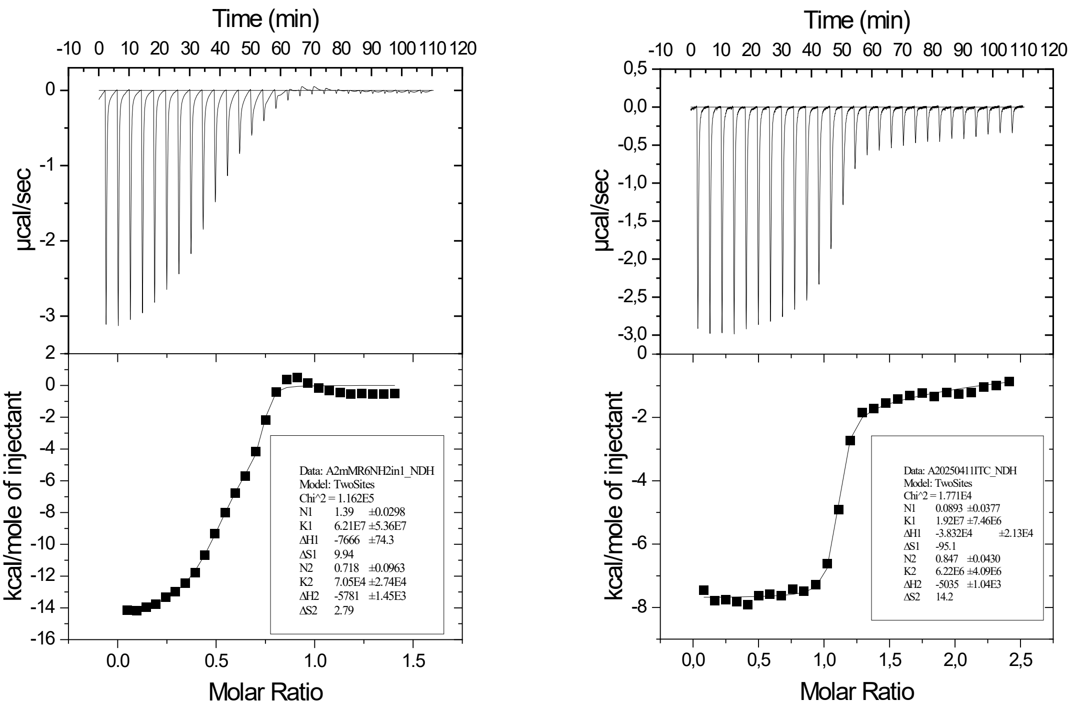

Figure S36. ITC direct binding titration of 1.2 mM CX4 into 20 µM H-(Arg)$_6$-NH$_2$ (left) and 100 µM H-(Arg)$_4$-NH$_2$ (right) in 10 mM NaH_2PO_4, pH 7.2, 25 °C.

References

[1] J. E. T. Corrie, C. T. Davis, J. F. Eccleston, "Chemistry of Sulforhodamine−Amine Conjugates" *Bioconjugate Chem.* **2001**, *12*, 186.

[2] J. E. T. Corrie, J. F. Eccleston, M. A. Ferenczi, M. H. Moore, J. P. Turkenburg, D. R. Trentham, "Ring-Chain Interconversion of Sulforhodamine-Amine Conjugates Involves an Unusually Labile C-N Bond and Allows Measurement of Sulfonamide Ionization Kinetics" *J. Phys. Org. Chem.* **2008**, *21*, 286.

[3] M. Stawikowski, G. B. Fields, "Introduction to Peptide Synthesis" *Curr. Protoc. Protein Sci.* **2012**, *69*, 18.1.1.

[4] G. B. Fields, R. L. Noble, "Solid Phase Peptide Synthesis Utilizing 9-Fluorenylmethoxycarbonyl Amino Acids" *Int. J. Peptide Protein Res.* **1990**, *35*, 161.

[5] L. A. Carpino, H. Shroff, S. A. Triolo, E.-S. M. E. Mansour, H. Wenschuh, F. Albericio, "The 2,2,4,6,7-Pentamethyldihydrobenzofuran-5-Sulfonyl Group (Pbf) as Arginine Side Chain Protectant" *Tetrahedron Lett.* **1993**, *34*, 7829.

[6] E. Kaiser, R. L. Colescott, C. D. Bossinger, P. I. Cook, "Color Test for Detection of Free Terminal Amino Groups in the Solid-Phase Synthesis of Peptides" *Anal. Biochem.* **1970**, *34*, 595.

[7] H. Kaur, P. Sharma, N. Patel, V. K. Pal, S. Roy, "Accessing Highly Tunable Nanostructured Hydrogels in a Short Ionic Complementary Peptide Sequence via pH Trigger" *Langmuir* **2020**, *36*, 12107.

[8] B. J. H. Kuipers, H. Gruppen, "Prediction of Molar Extinction Coefficients of Proteins and Peptides Using UV Absorption of the Constituent Amino Acids at 214 nm to Enable Quantitative Reverse Phase High-Performance Liquid Chromatography−Mass Spectrometry Analysis" *J. Agric. Food Chem.* **2007**, *55*, 5445.

[9] T. Wang, A. Riegger, M. Lamla, S. Wiese, P. Oeckl, M. Otto, Y. Wu, S. Fischer, H. Barth, S. L. Kuan, et al., "Water-Soluble Allyl Sulfones for Dual Site-Specific Labelling of Proteins and Cyclic Peptides" *Chem. Sci.* **2016**, *7*, 3234.

[10] Y. Tian, X. Zeng, J. Li, Y. Jiang, H. Zhao, D. Wang, X. Huang, Z. Li, "Achieving Enhanced Cell Penetration of Short Conformationally Constrained Peptides through Amphiphilicity Tuning" *Chem. Sci.* **2017**, *8*, 7576.

[11] A. Barba-Bon, Y.-C. Pan, F. Biedermann, D.-S. Guo, W. M. Nau, A. Hennig, "Fluorescence Monitoring of Peptide Transport Pathways into Large and Giant Vesicles by Supramolecular Host–Dye Reporter Pairs" *J. Am. Chem. Soc.* **2019**, *141*, 20137.

[12] A. Hennig, A. Hoffmann, H. Borcherding, T. Thiele, U. Schedler, U. Resch-Genger, "Quantification of Surface Functional Groups on Polymer Microspheres by Supramolecular Host–Guest Interactions" *Chem. Commun.* **2011**, *47*, 7842.

[13] S. Peng, A. Barba-Bon, Y.-C. Pan, W. M. Nau, D.-S. Guo, A. Hennig, "Phosphorylation-Responsive Membrane Transport of Peptides" *Angew. Chem. Int. Ed.* **2017**, *56*, 15742.
